# Keeping it complicated: Mitochondrial genome plasticity across diplonemids

**DOI:** 10.1038/s41598-017-14286-z

**Published:** 2017-10-26

**Authors:** Matus Valach, Sandrine Moreira, Steve Hoffmann, Peter F. Stadler, Gertraud Burger

**Affiliations:** 1 Department of biochemistry and Robert-Cedergren Centre for Bioinformatics and Genomics, Université de Montréal, 2900 Edouard-Montpetit, Montreal, H3T 1J4 QC Canada; 2 Leipzig University, LIFE - Leipzig Research Center for Civilization Diseases, Haertelstrasse 16-18, Leipzig, D-04107 Germany; 3 Bioinformatics Group, Department Computer Science, and Interdisciplinary Center for Bioinformatics, University Leipzig, Härtelstrasse 16-18, D-04107 Leipzig, Germany; 4 German Centre for Integrative Biodiversity Research (iDiv) Halle-Jena-Leipzig, Competence Center for Scalable Data Services and Solutions, and Leipzig Research Center for Civilization Diseases, University Leipzig, D-04107 Leipzig, Germany; 5 Max Planck Institute for Mathematics in the Sciences, Inselstrasse 22, D-04103 Leipzig, Germany; 6 Fraunhofer Institute for Cell Therapy and Immunology, Perlickstrasse 1, D-04103 Leipzig, Germany; 7 Department of Theoretical Chemistry of the University of Vienna, Währingerstrasse 17, A-1090 Vienna, Austria; 8 Center for RNA in Technology and Health, University of Copenhagen, Grønnegårdsvej 3, 1870 Frederiksberg C, Denmark; 9 Santa Fe Institute, 1399 Hyde Park Road, Santa Fe, NM 87501 USA; 10 Present Address: Department of Biochemistry and Molecular Biophysics, Columbia University, Hammer Health Science Center, 701 W 168th St, New York, NY 10031 USA

## Abstract

Chromosome rearrangements are important drivers in genome and gene evolution, with implications ranging from speciation to development to disease. In the flagellate *Diplonema papillatum* (Euglenozoa), mitochondrial genome rearrangements have resulted in nearly hundred chromosomes and a systematic dispersal of gene fragments across the multipartite genome. Maturation into functional RNAs involves separate transcription of gene pieces, joining of precursor RNAs via trans-splicing, and RNA editing by substitution and uridine additions both reconstituting crucial coding sequence. How widespread these unusual features are across diplonemids is unclear. We have analyzed the mitochondrial genomes and transcriptomes of four species from the *Diplonema/Rhynchopus* clade, revealing a considerable genomic plasticity. Although gene breakpoints, and thus the total number of gene pieces (~80), are essentially conserved across this group, the number of distinct chromosomes varies by a factor of two, with certain chromosomes combining up to eight unrelated gene fragments. Several internal protein-coding gene pieces overlap substantially, resulting, for example, in a stretch of 22 identical amino acids in cytochrome *c* oxidase subunit 1 and NADH dehydrogenase subunit 5. Finally, the variation of post-transcriptional editing patterns across diplonemids indicates compensation of two adverse trends: rapid sequence evolution and loss of genetic information through unequal chromosome segregation.

## Introduction

Genomes balance between stability and plasticity, i.e., between conservation and alteration of the genetic information. Genomic plasticity includes reshuffling (inversions, translocations, fissions, and fusions), and elimination and acquisition of DNA, collectively referred to as genome rearrangements. In bacteria, genome plasticity can change the expression level of certain genes, cause antigenic variation, and protect against invasion by phages and mobile elements^1^. Chromosome rearrangements in the nuclear genome contribute to speciation^2,3^. In certain taxa, rearrangements take place in a programmed manner during development^4–6^, while haphazard rearrangements drive progression of several human cancers (reviewed in^7^).

The large body of data on genome rearrangements in mitochondria documents not only sequence deletions, insertions, and reshuffling^8,9^, but also change of topology by linearization of the originally circular chromosome, as well as disintegration into multiple chromosomes^10–12^. A most spectacular kind of mitochondrial genome architecture is found in a group of unicellular marine flagellates: the diplonemids.

Traditionally, diplonemids (members of the Euglenozoa) have included two small genera, *Diplonema* and *Rhynchopus*. A recent addition is the genus *Hemistasia*, previously associated with Kinetoplastids, and containing a single recognized species, *H*. *phaeocysticola* ^13^. However, an environmental survey has uncovered two additional and diverse clades, named Deep-Sea Pelagic Diplonemids (DSPD) I and II^14^. Even more surprising are the recent data from the comprehensive Tara expedition, which classified diplonemids among the most cosmopolitan, most abundant, and most diverse eukaryotes in the oceans^15,16^. Yet, as of now, not a single species of the DSPD-clades has been isolated.

The first diplonemid whose mitochondrial DNA (mtDNA) has been characterized is the model species *Diplonema papillatum*. We discovered that all genes except one are fragmented in up to 11 pieces (modules) of about 40–550 nt length, and almost each gene piece resides on its own chromosome^17,18^. The fragmented structure of these mitochondrial genes is associated with a most unconventional architecture of the mitochondrial genome. This mtDNA consists of as many as 81 distinct circular chromosomes (each present in multiple copies) that fall into two classes based on molecule size (6 and 7 kbp) and sequence^19^. About 95% of a given chromosome’s length shares its sequence with that of the other members of its class. The remaining 5% of a chromosome is a distinctive, unique region, referred to as ‘cassette’, which includes a single gene module mentioned above^20^. Gene-content wise, however, the *D*. *papillatum* mtDNA is quite conventional, specifying proteins of the respiratory chain and oxidative phosphorylation, and the ever-present large and small-subunit ribosomal RNAs (mt-LSU rRNA, mt-SSU rRNA).

Gene fragmentation in *D*. *papillatum* mtDNA is compensated at the post-transcriptional level. Separately transcribed gene pieces are first trimmed to coding-only sequences (modules), and then modules belonging to the same gene are covalently joined (trans-spliced) to form a full-length mRNA or rRNA^21,22^. A number of mRNA and rRNA precursors is further decrypted by two different processes: (i) unique U-appendage RNA editing and (ii) substitution RNA editing at densely clustered sites, converting certain cytosines and adenosines into uridines and inosines, respectively (C-to-U; A-to-I)^18^. RNA editing by U additions also occurs in kinetoplastid mitochondria, but in that case, pre-mRNA is cut and resealed (reviewed in^23^), whereas in diplonemids, Us are attached to module ends. Further, organellar C-to-U RNA editing has been reported before, mostly in plants, but also in certain amoeba and fungi^24^. However, A-to-I substitutions in pre-mRNA and pre-rRNA are a first in diplonemid mitochondria.

Here, we investigate mitochondrial genome rearrangements across diplonemids, examining whether the eccentric, yet orderly mitochondrial genome structure of *D*. *papillatum* mitochondria is a landmark of diplonemids at large, or rather just one instance among a broad range of resourceful mtDNA architectures. We are also inquiring about the distribution and types of mitochondrial RNA editing across diplonemids. The present study is focusing on the *Diplonema/Rhynchopus* group, but also revisits data published by others on *Hemistasia phaeocysticola*.

## Results

**Figure 1:**
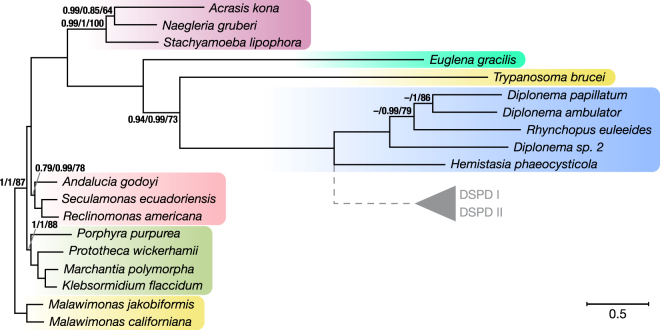
Phylogenetic relationships among diplonemids and other eukaryotes. The phylogenetic tree was constructed with Bayesian and maximum likelihood methods using 10 mitochondrion-encoded proteins. Unless indicated (Phylobayes/MrBayes/RAxML), posterior probabilities and bootstrap support values were 1 and 100%, respectively. The three methods yielded the same topology except within the *D/R* clade, where the branching order remained unresolved with Phylobayes (indicated by dashes); a broader taxon sampling will be required to reconstruct a topology with higher confidence levels. The bar shows the length corresponding to 0.5 substitutions per site. The position of the clade of uncultured diplonemids displayed in grey has been taken from trees based on nuclear 18S rRNA^14,16^. Colour-shading indicates major clades (from top to bottom): Heterolobosea, Euglenida, Kinetoplastida, Diplonemida, Jakobida, Archaeplastida, and Malawimonadida.

We have investigated mtDNAs from three diplonemid species available in culture collections, notably *D*. *ambulator*, *D*. sp. 2, and *R*. *euleeides* (Fig. 1). These species, together with *D*. *papillatum* examined earlier, represent the diversity of the *Diplonema/Rhynchopus* (*D/R*) clade according to nuclear-gene phylogenies^14,25^.

### The mitochondrial gene repertoire is conserved across diplonemids

Identification of gene modules encoded in diplonemid mtDNA is far from straightforward due to high sequence divergence and short module length. We used the following criteria: (i) sequence similarity with known mitochondrial genes and in particular with those from *D*. *papillatum*, (ii) evidence of directional transcription of the genomic region, (iii) inclusion in a mature transcript together with the more conserved module(s), and (iv) being flanked in the genome by the same repetitive motifs as those surrounding well conserved modules of a given mtDNA.

Using these criteria, we found in the mitochondrial genomes of the diplonemids investigated here the same 12 assigned genes present in *D*. *papillatum*, i.e., *atp6* (ATP synthase subunit 6), *cob* (apocytochrome b), *cox1*–*3* (cytochrome oxidase subunits 1, 2, and 3), *nad1*, *4*, *5*, *7*, and *8* (NADH dehydrogenase subunits 1, 4, 5, 7, and 8), *rnl*, and *rns* (large and small-subunit ribosomal RNA, mt-LSU and mt-SSU rRNA). Among the six unassigned mitochondrial genes (*y1-y6*) of *D*. *papillatum*, all but *y4* have been detected in the other diplonemids (Supplementary Fig. S1). Comparison of the inferred protein sequences of assigned mitochondrial genes shows rapid sequence evolution across diplonemids (Fig. 1). The cross-species identities range from moderate 46.6% for the most conservative protein Cox1 to merely 17.9% for the most variable, Nad5. With only ~9.5% overall identity, sequence conservation of the inferred Y proteins is extremely low.

### Marginal variation in module number and gene breakpoint positions

**Figure 2:**
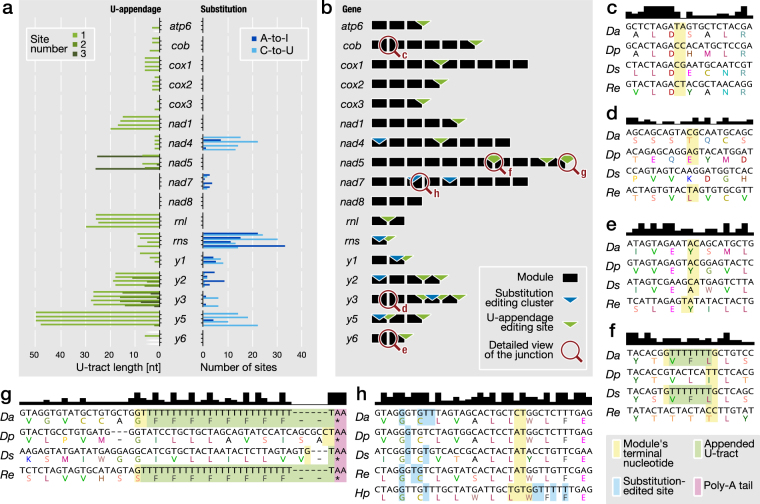
RNA editing sites and module junctions in diplonemid mitochondrial genes. (**a**) Number of RNA editing sites and added Us in mitochondrial transcripts of the four studied diplonemids. Gene names are indicated in the middle with sets of four bars to the left and right, whose height represents the corresponding number of appended Us (*left*) and substitution sites (*right*), respectively. Each bar of the set corresponds to one species, from top to bottom: *D*. *ambulator*, *D*. *papillatum*, *D*. sp. 2, and *R*. *euleeides*. Different green shades distinguish the various U-appendage editing sites in a given gene; unresolved sites are labeled with white fading bars. Dark and light blue bars indicate A-to-I and C-to-U substitutions, respectively. (**b**) Position of RNA editing sites relative to gene breakpoints. Inset, the feature key; the magnification glass symbol and letters refer to the close-up views of multiple cDNA sequence alignments in (**c**–**h**). (**c**–**h**) The upper track indicates by bar height the extent of sequence similarity. The inferred protein sequence is shown in the one-letter code below the nucleotide sequence. Inset, the colour-shading key for feature highlighting. Species name abbreviations: *Da*, *D*. *ambulator*; *Dp*, *D*. *papillatum*; *Ds*, *D*. sp. 2; *Re*, *R*. *euleeides*; *Hp*, *Hemistasia phaeocysticola*. (**c**) The well-conserved *cob*-m1 and -m2 junction. (**d**) The additional gene breakpoint in *y3*: *Ds_y3*-m1 corresponds to modules *y3*-m1 plus -m2 in other diplonemids. (**e**) A rare, minor junction shift in one of the species at the junction *y6*-m1 and -m2. (**f**) The internal U-appendage site between modules *nad5*-m7 and -m8 is shared by two out of four species. (**g**) The 3′ end of *nad5*-m11 is an example of U-tracts compensating for the size differences among the 3′ terminal modules. (In *D*. *ambulator* and *D*. *papillatum*, the module’s 3′ terminus was not determined precisely, i.e., the last nucleotide could be either genome-encoded, or appended to the transcript by RNA editing). (**h**) A-to-I and C-to-U editing sites in substitution clusters vary substantially between species; junction between *nad7*-m3 and -m4. (*H*. *phaeocysticola* contains an additional small substitution cluster; the exact location of module termini is currently unknown, but within the highlighted region).

In the four diplonemid species, the total number of modules that constitute the 17 shared mitochondrial genes is essentially identical (Table 1; Supplementary Table S1; Fig. 2b). The only exception is *y3* of *D*. sp. 2, which is composed of four instead of five modules, the first module corresponding to modules m1 and m2 in the other species (Fig. 2d). Further, across these taxa, homologous modules with high sequence conservation are identical in length. Size differences up to 31 bp occur in modules with divergent sequences (e.g., modules of *nad5* and *y1*-*y5*; Supplementary Table S1) and are almost all multiples of three in internal modules from protein-coding genes. Gene breakpoints are also highly conserved (Fig. 2c). Among the junctions that could be confidently aligned (59 out of 63 shared junctions), we found just seven cases with minor shifts (1 and 3 nt), each time in one of the four species (Fig. 2e, Supplementary Fig. S2).

**Table 1:** Mitochondrial chromosomes in *D/R* diplonemids.

Species	Multi-member classes of chromosomes^a^				Unclassified chromosomes^d^		Chromosomes with multiple modules^e^ (Nr. of modules)	Total chromosome count (Nr. of modules)
	Nr. of classes	Mono-module^b^	Multi-module	Multi-cassette^c^	Mono-module	Multi-module		
*D*. *papillatum*	2	78 (A,B)	3 (B)	—	—	—	3 (6)	81 (82)
*D*. *ambulator*	3	17 (A,B)	3 (A,B)	1(A) + 14(C)	1 + 1*	2 + 1*	21 (61)	40 (80)
*D*. sp. 2	4	59 (A–D)	4 (A,B)	n.d.	2	4	8 (21)	69 (79)
*R*. *euleeides*	4	56 (A–D)	9 (A–D)	n.d.	—	1	10 (24)	66 (80)

^a^The letters in parentheses indicate the chromosome classes in that category. Multimember classes are designated A, B, C, and D.

^b^Includes chromosomes, whose cassettes are lacking defined modules.

^c^Includes multi-module cassettes and mono-module cassettes. In *D*. *ambulator*, chromosomes of the C-class contain two cassettes, with c1- and c2-cassette associations known for four chromosomes (see also Supplementary Table S2 and Supplementary Fig. S1).

^d^Asterisk indicates ‘hybrid’ chromosomes, composed of a classified class-like moiety and a unique moiety (see also Fig. 3c).

^e^Includes chromosomes with multiple modules arranged either in a single cassette or array, or in two cassettes or arrays. n.d., not determined.

### Number of mitochondrial chromosome classes differs among diplonemids

In the present study, we have obtained about 200 kbp of random mtDNA sequence for each, *D*. *ambulator*, *D*. sp. 2, and *R*. *euleeides*. Sequence assembly yielded four complete chromosomes from *D*. sp. 2, together with partial chromosomes sequences from all species (Supplementary Table S2). Despite the high coverage (>100×), sequence assembly of complete chromosomes is often hampered because of numerous intra- and inter-chromosomal repeats. Nevertheless, the new data confirm earlier reports that mitochondrial chromosomes in diplonemids are circular molecules of ~4–10 kbp in length^19,26,27^.

**Figure 3:**
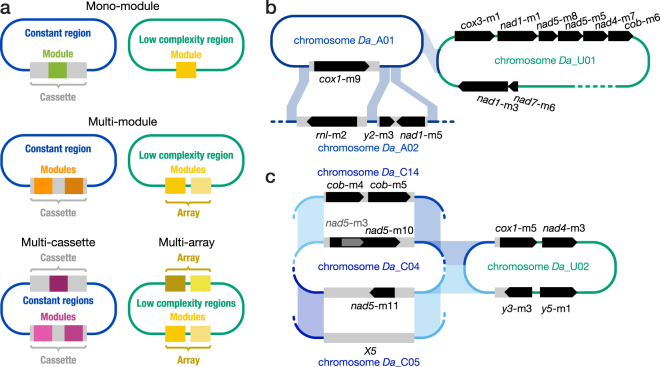
Mitochondrial chromosome architecture in *D/R* diplonemids. Thin blue and green lines indicate non-coding regions of classified and unclassified chromosomes, respectively. Dashes symbolize incomplete sequence. Grey bars specify cassettes. Modules are represented by coloured squares or black pointed bars (the tip indicating the direction of transcription). (**a**) Scheme of the various module/cassette arrangements including single or multiple modules per cassette and single or multiple cassettes per chromosome (*left*). When chromosomes lack recurrent cassette-framing motifs shared with other chromosomes, cassette start and end positions cannot be defined (*right*; for details, see Table 1 and Supplementary Table S2). (**b**,**c**) Members of the same chromosome class share a pair of >100 bp-long cassette-flanking sequence motifs. Chromosomes lacking recurrent cassette-framing motifs are categorized as ‘unclassified’ (U-chromosomes). (**b**) *D*. *ambulator*’s A-class chromosomes and similarity to the unclassified chromosome *Da*_U01, which has the largest module array. Note that the second cassette in the chromosome *Da*_A02 interrupts one of the two conserved cassette-flanking motifs. (**c**) *D*. *ambulator*’s C-class chromosomes and similarity to the unclassified chromosome *Da*_U02. C-class chromosomes contain two cassettes, a c1- and a c2-cassette (top and bottom, respectively) facing each other and each surrounded by a pair of specific recurrent cassette-framing motifs. The association of c1- and c2-cassettes in incompletely sequenced chromosomes was confirmed by PCR and amplicon sequencing in four cases (for details, see Supplementary Table S2). Note that the cassette *X5* of the chromosome *Da*_C05 does not contain a recognizable module. The chromosome *Da*_U02 (GenBank nr. JF698652) shares one of the two inter-cassette regions with C-class chromosomes; therefore, only one end of its two cassettes/arrays can be pinpointed.

For each of the three species, we aligned the sequences that surround gene modules. This allowed us to precisely locate module-adjacent unique regions constituting the distal portions of cassettes and, in addition, to identify recurring sequence motifs, which form the ‘constant regions’ that are shared with other chromosomes (see Fig. 3a). Then, chromosomes with similar constant regions were grouped together in a class (see Methods), revealing one or two classes more than in *D*. *papillatum* (Table 1; classes are referred to as *Da*_A, *Da*_B, etc.) There is no significant sequence similarity among the constant regions of the various classes from different species.

MtDNA of *D*. *ambulator* and *D*. sp. 2 also includes chromosomes of unique structure, which constitute the ‘unclassified’ (U) category (Table 1; Fig. 3a). Since these chromosomes do not share module-adjacent motifs with any other contig, cassette start and end points remain undefined. The four completely assembled chromosomes in *D*. sp. 2 belong to this category.

### Diverse module/cassette arrangements in mitochondrial chromosomes across diplonemids

Unexpectedly, the three diplonemids investigated here do not adhere to the orderly mitochondrial chromosome architecture seen in *D*. *papillatum*—an architecture characterized by one module per cassette and one cassette per chromosome. In mtDNA of *D*. *ambulator*, *D*. sp. 2, and *R*. *euleeides*, we encountered, in addition to the ‘orthodox’ configuration (e.g., Fig. 3b, chromosome *Da*_A01), multiple modules congregated in a cassette, as well as, though less abundant, chromosomes harboring multiple cassettes (Table 1; Fig. 3a).

The two newly encountered architectures are most abundant and pronounced in *D*. *ambulator*. An example of a multi-module cassette resides in chromosome *Da*_A02, enclosing the third module of *y2* (*y2*-m3) and *nad1*-m5 in opposite orientations (Fig. 3b). Multiple modules are also present in certain chromosomes of the ‘unclassified’ category and are arranged in cassette-like arrays. The largest array occurs in the chromosome *Da*_U01, containing as many as six modules spaced by merely ~100 bp of non-coding sequence (Fig. 3b). Throughout the diplonemids, modules grouped together in multi-module cassettes or arrays belong to different genes and are randomly combined. Only in one case are two such modules also neighbors in the mature transcript (chromosome *Da*_C14; Fig. 3c), and only one module combination occurs in two different species, namely *nad7*-m6 and *y2*-m3 in *D*. *papillatum* and *D*. sp. 2.

*Da*_A02 is a multi-cassette chromosome, whose configuration was readily resolvable by sequence assembly, because the two cassettes are only ~200 bp distant from one another (Fig. 3b). However, when cassettes on a chromosome are separated by long repeat stretches, assembly will likely result in several disconnected contigs. We confirmed four such instances by PCR, showing that contigs carrying a cassette c1 are part of a chromosome that also holds a cassette c2 in opposite position on the circular molecule (e.g., chromosome *Da*_C04; Fig. 3c). We infer that *D*. *ambulator* mtDNA contains 14 bi-cassette C-class chromosomes (Table 1). The unclassified chromosome *Da*_U02 (GenBank n. JF698652) has a similar cassette-like arrangement, but differs from C-class chromosomes in half of its non-coding sequence (Fig. 3c).

Finally, we found cassettes with tentative orphan modules in chromosomes of *D*. *ambulator* and *D*. sp. 2. Akin to *X12* and *X18* of *D*. *papillatum*, these hypothetical modules are transcribed bi-directionally at low level. Transcripts have variable termini and apparently are not post-transcriptionally edited, polyadenylated, or joined to other modules. In *D*. *ambulator*, all six orphan cassettes are short (~94 bp), share high nucleotide sequence identity to each other (86%), and reside on C-class chromosomes (Fig. 3c; Supplementary Table S2). Whether they have a biological function is currently unknown.

### Certain modules code for pieces of two different genes

In the four diplonemids, about a third of the modules in multi-module cassettes are overlapping, either on the same strand or on opposite strands (Supplementary Figs S1, S3). Overlapping modules are exclusively pieces of protein-coding genes. In each of the three newly examined diplonemids, we encountered modules that are entirely nested one within the other (e.g., *nad5*-m3 embedded in *nad5*-m10 of the chromosome *Da*_C04 in Fig. 3c; Supplementary Fig. S3). All nested modules that are encoded on the same strand have the same reading frame. This results in two differently processed module transcripts that are incorporated into distinct mRNAs, yielding up to 22 amino acid-long identical sequences in two different proteins. In all cases, the embedded, shorter module is more divergent in protein sequence than the enclosing, larger counterpart (Table 2, Supplementary Fig. S3). As we discuss in more detail below, this may indicate that the enclosing ‘host’ module has secondarily shared its sequence with another gene.

**Table 2:** Embedded modules code for more divergent protein regions than their enclosing counterparts.

Strand	Module overlap size [aa (bp)]^a^	Enclosing module [Embedded module]		
		Module name	Nr. conserved residues^b^	% mean pairwise identity^c^
Same	19 (57)	*nad5-*m10 [*nad5-*m3]	3 [0]	29.2 [11.6]
	22 (66)	*cox1-*m9 [*nad5-*m3]	1 [0]	22.1 [11.6]
	19 (57)	*y2-*m3 [*y2*-m4]	7 [0]	51.2 [13.0]
	18 (56)	*cob-*m5 [*y5*-m3]	1 [1]	30.6 [22.5]
Opposite	11 (56*)	*y3-*m3 [*y5-*m3]	2 [1]	30.7 [22.5]
	27 (94*)	*nad4-*m1 [*y1-*m2]	3 [0]	21.4 [13.1]

Values were calculated based on the MSAs of proteins from *D*. *ambulator*, *D*. *papillatum*, *D*. sp.2, and *R*. *euleeides*. The analyzed region corresponds to the embedded module. For details, see Supplementary Fig. S3.

^a^Size of the overlapping stretch between enclosing and embedded module at the protein and DNA level; aa, amino acids; bp, base-pairs. Asterisks indicate that the overlap size includes the coding part, as well as the 3′ UTR of the embedded module.

^b^Number of amino acid residues conserved in the analyzed region across the four species.

^c^Percentage of mean pairwise identity in the analyzed region across the four species.

### U-appendage RNA editing: shared and species-specific sites

When comparing mitochondrial transcript and genome sequences of the three diplonemids studied here, we observed that RNA-DNA-indel differences consist exclusively of T insertions (in cDNA). As in *D*. *papillatum*, these insertions represent up to ~50-nt long U-additions at module-transcript 3′ ends, and occur in protein-coding and rRNA genes (Fig. 2a,b; Supplementary Table S3). About half of the 21 U-appendage sites are shared among all four diplonemids.

Only two sites (in *cox1* and *nad4*) have identical U-tract length across the four *D/R* taxa. Remarkably, cross-species length variation of the U-tract at all other sites coincides with size deviation of the module upstream of the Us. For example, in *D*. *ambulator* and *R*. *euleeides*, the 3′-terminal *nad5* module (*nad5*-m11) is about 25 nt shorter than those of *D*. sp. 2 and *D*. *papillatum*. In the former two species, RNA editing appends about 26 Us, but in the latter two taxa, only one or none (Fig. 2g). At internal modules of protein-coding genes, cross-species variations of U-tract length respect the corresponding reading frame (e.g., Fig. 2f; Supplementary Table S6). Apparently, U-appendage compensates for sequence loss in gene modules, as proposed earlier^24^.

### Substitution RNA editing: same cluster positions but different sites

The above-mentioned screening for mitochondrial RNA-DNA differences in the three diplonemids examined here also revealed nucleotide substitutions, notably A-to-I and C-to-U RNA editing as in *D*. *papillatum* ^18^. We experimentally confirmed the presence of inosines for mt-SSU rRNA of *D*. *ambulator* (Supplementary Fig. S4). The total number of substitution sites ranges from 78 (*D*. sp. 2) to 114 (*D*. *papillatum*) (Supplementary Table S3).

All these RNA editing sites are arranged in compact clusters (Fig. 2a,h; Supplementary Fig. S1). Out of the seven clusters in *D*. *papillatum*, five (in *nad4*, *nad7*-m3, *rns*, *y2*, and *y5*) are conserved across the four *D/R* group diplonemids, whereas those of *y1* and *y3* are only present in *D*. sp. 2 and *R*. *euleeides*, respectively. The latter two species share an additional small editing cluster in *nad7*-m5. Cluster sizes vary considerably, and individual editing sites within clusters only rarely coincide throughout the four species (Fig. 2h; Supplementary Fig. S1). As demonstrated earlier for *nad4*, substitution RNA editing makes protein sequences more similar within diplonemids and to homologs of other eukaryotes^18^.

We hypothesized that substitution editing equalizes the genomic A + T-content differences across diplonemids. Yet, this is only the case for mt-SSU rRNA, with a 16% across-diplonemid A + T-content difference among *rns* gene sequences shrinking to 7% among the edited transcripts. Interestingly, the A + T-content difference of diplonemid mt-LSU rRNA, which does not undergo substitution editing, is also 7%. The leveling of nucleotide composition in mt-SSU rRNA is due to predominant A-to-I substitutions in A + T-rich mtDNAs (*R*. *euleeides*) and predominant C-to-U edits in A + T-poor mtDNAs (*D*. *papillatum*; Fig. 2a). Apparently, secondary structure stability of the mature molecule favors the window of 51–58% A + T for diplonemid mt-rRNAs.

### *Hemistasia* revisited: much more of the same compared to *D/R* diplonemids

A recent investigation of four mitochondrial genes (*cob*, *cox1*, *cox2*, and *nad7*) from *Hemistasia phaeocysticola* has shown that this apparently fast-evolving diplonemid^13^ has all particularities observed before in *D*. *papillatum*: multi-partite mtDNA, fragmented mitochondrial genes, and U-appendage, as well as C-to-U and A-to-I substitution RNA editing^28^. Most gene breakpoints present in these four genes in the *D/R* diplonemids are found at same positions in *H*. *phaeocysticola*. Even in cases where junctions are ambiguous and remain to be experimentally confirmed in *Hemistasia*, potential shifts are only minor (e.g., Fig. 2h).

**Figure 4:**
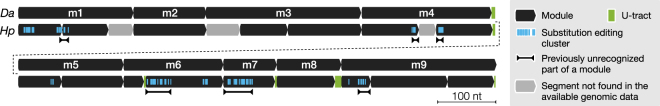
Substitution editing clusters, U-appendage sites, and gene breakpoints in *cox1* of *Hemistasia phaeocysticola* (*Hp*) that are absent from *cox1* of *D*. *ambulator* (*Da*) and the other three *D/R*-group diplonemids.

However, these four *Hemistasia* genes are much more fragmented than those from the *D/R* clade. For example, the *Hp_cox1* gene includes according to the authors, in addition to 17 average-sized modules (29–132 bp), seven miniature modules (down to 7 bp), which are only seen in the transcript, but could not be identified in the available genome sequence^28^. However, our data reanalysis leads to another conclusion. Most of these postulated mini-modules can be explained by large A-to-I and C-to-U editing clusters that impede proper alignment of mature transcript and genomic sequences, and thus hamper pinpointing gene-module starts and ends. For instance, at the gene breakpoint corresponding to the junction *cox1*-m6/m7 in the *D/R* group species, the postulated mini-module *cox1*-m18 of *Hemistasia* is in fact the 5′-moiety of the downstream module, camouflaged by heavy substitution RNA editing (Fig. 4; Supplementary Fig. S5). Larger modules reported as missing in the *Hemistasia* mtDNA are most likely encoded by chromosome classes that have not been sampled by the targeted PCR approach. To summarize, there are almost twice as many gene fragments in *H*. *phaeocysticola* mtDNA of about half the length compared to the *D/R* diplonemids (Supplementary Fig. S6). In fact, all modules larger than ~130 bp in the *D/R* taxa are split into two or more pieces in *Hemistasia*.

Our reexamination of the published *Hemistasia* data also allowed us to examine the cross-species distribution of substitution editing clusters. All four genes analyzed in *Hemistasia* appear to undergo substantial deamination RNA editing in that species, but not *cob*, *cox1*, and *cox2* in the four *D/R* diplonemids (Supplementary Fig. S7). The two editing clusters present in *nad7* of the *D/R* clade (Fig. 2b) are also found in *Hemistasia*, but the latter species contains 10 additional clusters with a total of 34 sites (Fig. 2h; Supplementary Fig. S7). Intriguingly, most additional substitution RNA editing clusters in *Hemistasia* occur near *Hemistasia*-specific module junctions, as does half of the U-appendage sites (Fig. 4; Supplementary Fig. S7).

Finally, the allelic state of the mitochondrial genomes appears to differ between that of *D/R*-diplonemids and *Hemistasia*. In contrast to an almost exclusively bi-allelic mode of single nucleotide polymorphic (SNP) sites in *D*. *papillatum* ^18^ and the three species investigated here (frequency of ~0.05–0.25% SNP per bp of coding sequence), *Hemistasia*’s *cox1* gene modules were reported to contain ~7.4% SNPs with up to four different alleles^28^. Unless the culture used in the study is not monoclonal, mitochondrial sequence heterogeneity of this diplonemid appears to be considerably higher than that of the *D/R* clade. Interestingly, certain SNP sites in *Hemistasia* undergo substitution RNA editing with the result that the inferred protein isoforms are more similar to one another (Supplementary Fig. S5C). This is consistent with our observation that substitution RNA editing renders diplonemid proteins more similar to homologs in other eukaryotes.

## Discussion

### Plasticity of the multipartite genome architecture in diplonemid mitochondria

We show here that in the *D/R* diplonemids, the configuration and module content of mitochondrial chromosomes vary considerably. First, the number of multi-member chromosome classes ranges from two to four. Second, some chromosomes (the ‘U’ category) are ‘outsiders’, as they do not cohere to any class. Third, in contrast to the regular architecture in the type species *D*. *papillatum*, mitochondrial chromosomes of the other three diplonemids may contain one or two cassettes (or module arrays) instead of only one, with ~15% of the cassettes including multiple modules (Table 1). Finally, when multiple gene pieces reside on the same chromosome, they seem grouped together at random, reflecting a high rate of genome rearrangement.

Multipartite organellar genomes are generally characterized by long repeat sequences that are shared by all chromosomes (e.g., in mitochondria of many metazoans^29^, green algae^30^, and protists^31^), or in plastids of dinoflagellates^32,33^). A notable exception are the multiple unique chromosomes, as well as low-number member classes discovered in *D*. *sp*. 2 and *R*. *euleeides*, possibly indicating ongoing birth and death of chromosome classes. For instance, the chromosomes *Ds*_U02 and *Ds*_U03 might be in the process of founding a new common class, as they share two short regions (~100 and ~300 bp) of almost identical sequence. In contrast, the chromosomes *Da*_U01 and *Da*_U02 could be on their way to become members of the large classes, as they share some sequence similarity (~1 kbp with A and B classes, and ~2 kbp with the C class, respectively; Fig. 3). Alternatively, these chromosomes may be drifting away from their earlier affiliation with the larger classes.

### Selection pressure imposed by multipartite genomes

When a genome is fragmented, there are essentially two ways to avoid gene loss during cell division: a well-organized partitioning system or, alternatively, stochastic repartition of massively and evenly amplified DNA. Cell microscopy studies of diplonemids show no indication of a kinetoplast body^19,26^, a structure that ensures equal subdivision of the multipartite trypanosomatid mtDNA between daughter cells (reviewed in^34^). The up to 130-fold variation in copy numbers of diplonemid mitochondrial chromosomes (correlating neither with a chromosome class, nor a particular gene; Supplementary Fig. S8; Supplementary Table S2), and the unusually high amount of mtDNA per cell (1:1 weight ratio of nuclear DNA versus mtDNA in *D*. *papillatum*) indicate a stochastic mode of mtDNA segregation and/or replication in diplonemids. This situation makes it quite plausible that certain chromosomes, and thus modules, are lost along generations. Therefore, a reduction in chromosome number via including multiple modules per circle instead of just one could provide an adaptive advantage.

### Gene fragmentation allows multi-purpose use

Sequence overlaps of adjacent genes have been documented in numerous mitochondrial genomes across the eukaryotic tree (e.g., see^35,36^). Diplonemids, however, take gene overlaps to a next level by reusing gene segments. One of the most spectacular cases involves *nad5* and *cox1* from *D*. sp. 2, where module *nad5*-m3 is completely embedded in *cox1*-m9 (Supplementary Fig. S3). This results in identical subsequences in the Nad5 and Cox1 proteins. Structural alignments of the deduced diplonemid proteins to homologs with known three-dimensional structure^37–39^ indicate that all dual-purpose segments correspond to peripheral loops and short α-helices that connect trans-membrane helices, i.e., to generalizable domains of membrane proteins (Supplementary Fig. S9). We posit that a diplonemid mitochondrial genome with even smaller mitochondrial gene fragments would contain a larger number of dual-purpose modules than seen in this study, because the shorter the fragment, the more likely can the sequence it encodes be used for functionally different proteins—a hypothesis that would be worthwhile testing in *Hemistasia*.

The use of a given module transcript for distinct mRNAs observed in diplonemid mitochondria is equivalent to alternative trans-splicing in the metazoan nucleus (reviewed in^40,41^). The difference is in the outcome: alternative splicing increases proteome complexity, whereas gene-module reuse results in the net reduction of coding sequence, indicating different evolutionary pressures.

### U-appendage RNA editing restores missing coding sequence

Most gene breakpoints are precisely conserved across the four *D/R*-clade diplonemids, but the length of homologous modules can vary considerably when gene regions are poorly conserved. For example, the 3′-terminal gene module *nad5*-m11, which specifies a functionally important trans-membrane helix of the Nad5 protein^39^, is about 25 nt shorter in *D*. *ambulator* and *R*. *euleeides* compared to the other two species. The missing coding sequences are added post-transcriptionally in the form of a U-tract of the corresponding length, which gives rise to a phenylalanine tract in the protein (Fig. 2g).

A similar restoration of incomplete genes occurs during maturation of tRNAs in the eukaryotic nucleus and many archaea and bacteria and involves addition of a CCA sequence at the transcript’s 3′ end (reviewed in^42^). The completion of 3′ termini of several animal mitochondrial tRNAs by polyadenylation represents another well-known example of post-transcriptional ‘repair’^43,44^. Lastly, in certain dinoflagellate mtDNAs, the gene *cox3* is split into two fragments, which are brought together at the RNA level by an unknown mechanism^45^. This trans-splicing is accompanied by insertion of an oligo-A tract^46^, thus patching the missing information similar to U-appendage editing in diplonemids.

### Module fusion or fission?

The breakpoints of *D/R*-clade mitochondrial genes are all identical, with one exception. The *y3* gene of *D*. sp. 2 is lacking one split (Fig. 2d), i.e., the first ~300 nt of the corresponding mRNA are specified by a single gene module, but by two modules in the other *D/R* species. A comparison across all diplonemids shows that half of the *Hemistasia* gene modules are fused in the *D/R* species. *Hemistasia* might have diverged more rapidly than the *D/R* clade by accelerating gene fragmentation^28^. In this case, the gene structure in the *D/R* group would represent a more ancestral state. Alternatively, the extreme gene fragmentation seen in *Hemistasia* may have already reigned in the common ancestor of all diplonemids, implying that the larger modules sizes in the *D/R* clade emerged by fusion of smaller gene pieces.

### Evolutionary trends and strategies in diplonemid mitochondria

Under the premise of adaptive evolution, module nesting and U-appendage RNA editing in diplonemid mitochondria would be strategies to patch losses of coding sequence. Reduction of the genetic information would result in a more compact genome, thus countering the effect of unequal chromosome segregation. However, from the perspective of constructive neutral evolution^47,48^, stochastic chromosome loss during segregation and ongoing gene decay were tolerated and allowed to accumulate because of the pre-existing, sophisticated post-transcriptional machinery.

According to nuclear rRNA phylogenies^13,14,16,49^, the two DSPD clades evolve at a slower pace than the *D/R* clade and the *Hemistasia* group in particular. DSPD species might therefore provide a unique window on earlier stages of mitochondrial innovations in diplonemids. Work is underway to isolate and culture representatives of these environmentally and evolutionarily intriguing taxa.

## Materials and Methods

### Strains, culture, and DNA and RNA extraction

*Diplonema papillatum* (ATCC 50162), *D*. *ambulator* (ATCC 50223), *Diplonema* sp. 2 (ATCC 50224), and *Rhynchopus euleeides* (ATCC 50226) were obtained from the American Type Culture Collection and cultivated axenically as described earlier^22,27^. Total cellular nucleic acids were extracted with a home-made Trizol substitute^50^, allowing enrichment in small circular molecules, including diplonemid mitochondrial chromosomes^51^. DNA and RNA for library construction and other manipulations were obtained from the same sample. Residual RNA in DNA preparations was removed by RNase I treatment and samples were cleaned up using Genomic-tip 100/G (Qiagen). Residual DNA in RNA preparations was removed by either RNeasy (Qiagen) column purification, or digestion with TURBO DNase (Invitrogen) followed by extraction with the Trizol substitute.

### Reverse transcription, PCR, 5′ RACE, RNase cleavage of glyoxalated RNA, and primer extension

Reverse transcription was performed with the AMV reverse transcriptase (Roche) or SuperScript IV Reverse Transcriptase (Thermo). DNA was amplified with Q5 High-Fidelity DNA Polymerase (New England BioLabs) or Platinum SuperFi DNA polymerase (Thermo). PCR products were purified using the Wizard SV Gel and PCR Clean-Up system (Promega) or Monarch DNA Gel Extraction Kit (New England BioLabs). To map the 5′ end of transcripts (5′ RACE), the RNA adaptor-oligonucleotide dp124 was ligated to total RNA with RNA ligase 1 (New England BioLabs) in the presence of 1 mM ATP and 15% PEG8000 for 4 h (1 h at 25 °C, 1 h at 16 °C, 2 h at 4 °C); RNA was then extracted and reverse-transcribed as described above. Inosines were mapped by primer extension on glyoxal-treated and RNase T1-digested RNA as described previously^18^. Detailed experimental procedures are available at https://www.protocols.io/u/matus-valach. Oligonucleotides (purchased from BioCorp and IDT) that were used as primers and adaptors are listed in Supplementary Table S4.

### Genome and transcriptome sequencing and assembly

Illumina Tru-Seq genomic paired-end libraries were constructed from total DNA, multiplexed, and sequenced in a single MiSeq lane. Adaptor removal and quality clipping of reads were carried out as described previously^18^. Mitochondrial and nuclear genomic contigs were assembled from total DNA reads using Spades v3.8.0^52^. Total RNA-Seq libraries were constructed using an Illumina TruSeq kit, multiplexed, and sequenced in one MiSeq and one HiSeq lane. For *de novo* assembly, we used Trinity 2.2.0 with default parameters^53^. Both library construction and sequencing of DNA and RNA were outsourced to the Genome Quebec Innovation Centre. Details on technology, read counts, and length are compiled in Supplementary Table S5. All data were deposited at NCBI: assembled mitochondrial genomic contigs and transcripts in GenBank (MF436742-MF436981; for details, see Supplementary Tables 2 and 7), and raw sequence data under BioProject PRJNA392339 (for accession numbers, see Supplementary Table S5).

### Module and cassette annotation

*Initial annotation*. In general, *de novo* assembly of RNA-Seq reads produced partial mitochondrial transcripts because of the substantial amount of precursors in RNA preparations hampering full assembly. Transcripts were identified in two ways: by aligning them against known mitochondrial proteins, and in particular those of *D*. *papillatum*, using exonerate (–model protein2genome)^54^, and by HMMER searches (HMMER 3.0)^55^ against profile Hidden Markov Models (HMMs) constructed from excavate proteins. An in-house Python script (mitoTranscriptomeReconstruction.py; available upon request), based on the Overlap-Layout-Consensus (OLC) algorithm, compares the identified transcript fragments to each other with BLAT v. 35 × 1^56^ and aligns identical regions. The script specifically tolerates poorly aligning transcript ends that correspond to the 5′ and 3′ flanking regions of unprocessed modules. To identify mitochondrial contigs and to delimit modules, full-length transcripts were reconstructed semi-manually and then compared with genomic contigs using BLAT. *Identification of additional cassettes*. We used the non-coding (or non-module) regions of initially identified mitochondrial contigs to search similar sequences in other contigs with NCBI-BLAST v2.3.0 (default options)^57,58^. Among the contigs with significant hits, those with a length >200 bp and coverage >100× were considered mitochondrial candidate contigs. Occasionally, we detected chimeric contigs (part nuclear, part mitochondrial), which were produced by a single chimeric read pair. Such chimeric contigs were split to keep the mitochondrial and discard the nuclear moiety. Split point definition was based on their uneven coverage, since the coverage of validated mitochondrial contigs was >200× (Supplementary Table S2), whereas that for validated nuclear contigs was by far lower (~5× for unique and <100× for repetitive sequences). Mean coverage of a cassette or module array (modules + 50 bp up- and downstream) was calculated after mapping DNA-Seq reads onto mitochondrial contigs with Bowtie2 in the local mode. Prior to mapping, reads were merged with BBMerge (rem k = 62 extend2 = 50 ecct) and deduplicated with Dedupe (ac = f) from the BBMap 36.99 suite (http://jgi.doe.gov/data-and-tools/bbtools/). Box plots and data-point distributions were generated using BoxPlotR (http://shiny.chemgrid.org/boxplotr/)^59^. *Search for initially unrecognized modules and genes*. Missing terminal modules were uncovered by iterative mapping of RNA-Seq reads on a partially reconstructed transcript reference and reads extending beyond the reference but not containing flanking regions were used for extending the partial transcript. In these cases, we confirmed the structure of the mature transcript by RT-PCR with an oligo-dT primer (for details, see above) and by sequencing the PCR amplicon. To pinpoint the highly divergent *Y* genes in the three diplonemids studied here, we first performed BLAST searches with DNA and inferred protein sequences of the *D*. *papillatum Y* genes against the *de novo* assembled transcripts and candidate mitochondrial genomic contigs. Once a *Y* gene’s most conserved module was spotted, the full transcript was reconstructed using the script mitoTranscriptomeReconstruction.py or by the aforementioned iterative mapping. Alternatively, we mapped the RNA-Seq data with Bowtie2^60^ in local mode on the candidate mitochondrial genomic contigs, extracted soft-clipped sequences, and searched for those reads and read pairs that span two (or more) contigs. Finally, homologous *Y* gene modules were used to build HMM profiles to screen species in which certain modules were not identified with the above procedures. In unresolved cases, we used 5′ RACE (see above) and amplicon sequencing to precisely map the 5′ end of the mature transcript (e.g., *y3* in *D*. sp. 2, *R*. *euleeides*, and *D*. *papillatum*). Pairwise nucleic acid and protein identities and similarities were calculated from a multiple sequence alignment, using the IUB and BLOSUM62 weight matrix, respectively, and visualized in Geneious 9.1^61^. *Inference of module junctions*. Out of the 251 module junctions across the four diplonemids investigated here, the exact position of 115 could not be inferred from the transcript sequence alone because of identical nucleotides at the 3′ end of the upstream module and the 5′ end of the downstream module (up to 6 nt identity). However, the inspection of mapped reads allowed us to precisely position the junction in most cases. The remaining 11 uncertain junctions are listed in Supplementary Table S6; all are adjacent to long U-appendages (two in *D*. *papillatum*, *D*. *ambulator*, and *D*. sp.2, and five in *R*. *euleeides*). In these cases, we chose the position downstream of a genome-encoded T residue. Similarly, when the last module ends with a T or an A, the residue could be genome-encoded or originate from U-appendage or polyadenylation, respectively. In cases where disambiguation was not feasible, we chose the genome-encoded base to represent the terminal nucleotide. *Errors at module junctions*. In contrast to earlier small-scale analyses^21^, the high coverage of our RNA-Seq data allowed us to detect a small number of imprecise trans-splicing events at certain junctions (usually <1%, but, for example, up to ~15% at the junction *Da_y2*-m3/m4). Four types of errors were noted: (i) over- or under-trimming of nucleotides at either the 5′, or 3′ end participating in the module-joining (usually <3 nt); (ii) U-addition at a 3′ end normally not undergoing U-appendage RNA editing (usually <3 nt); (iii) incompletely trimmed 3′ end instead of a U-tract; and (iv) mis-joining of non-cognate modules (usually <1%). These errors apparently represent the inherent noise of biological processes. An in-depth, comprehensive investigation of these phenomena is in preparation.

### Chromosome classification

In *D*. *papillatum*, a cassette is defined as a unique sequence flanked by recurring motifs of the class-specific constant region (Fig. 3a). To classify mitochondrial chromosomes of the other diplonemids, module-containing contigs were compared among each other by BLAST to identify common sequences upstream and downstream of modules. Cassette-flanking motifs were defined as sequences 5′ or 3′ adjacent to modules, having >90% identity over >100 bp, and being shared by the highest number of contigs. Contigs with the same pair of cassette-flanking motifs are considered (partial) chromosomes of the same class. Cassette starts and ends were precisely determined by aligning these contigs using MAFFT v7.222 (–auto–adjustdirection)^62^, and placing boundaries at positions where sequence identities dropped below 90% over 100 bp.

### Detection of RNA editing

Mapping of DNA-Seq reads onto mitochondrial contigs was performed with Bowtie2 in local mode, while RNA-Seq reads were mapped with segemehl^63^ (available at http://www.bioinf.uni-leipzig.de/Software/segemehl-diplonema/). The latter tool allows correct mapping of intensely edited regions using a reduced alphabet during the extension step (-F 6), with an accuracy threshold of 95% (-A 95). Reads aligning with indels were discarded. In order to detect substitution editing sites, DNA and RNA variants were called with the GATK tool (-T UnifiedGenotyper -ploidy 100 -glm BOTH -stand_emit_conf 30 -stand_call_conf 30 -read_filter MappingQuality -drf DuplicateRead). DNA variants were then subtracted from RNA variants with the vcf-isec utility from the vcftool suite v0.1.12b. Variants were further filtered with the GATK tool (-T SelectVariants) according to the criteria “StrandOddsRatio” (SOR < 3), which detected the strand bias (only for DNA), and “QualByDepth” (QD > 2.0), which selected regions with sufficient mapping quality and coverage. Finally, variants were visually inspected. Incorrectly called variants were removed (<5%, occurring at module junctions due to read mapping artifacts), whereas variants with independent support were added (<1%, RNA editing sites that coincide, for example, with a genomic indel). Editing by U-appendage was detected by performing a local mapping of DNA-seq and RNA-seq reads onto reconstructed RNA transcripts with Bowtie2 and Segemehl, respectively. Regions not covered by DNA-Seq reads but overlapped by poly-T sequences on RNA-Seq reads specify loci of U-appendage. No other type of insertion editing was identified. The GenBank accession numbers for sequences of edited RNAs are listed in Supplementary Table S7.

The long conserved U-homopolymer downstream of *y5-*m1 could not be fully resolved by Illumina sequencing reads alone, as observed previously^18^. To more precisely measure its size, we performed RT-PCR with a high-fidelity DNA polymerase, and sequenced the amplicons from both sides of the U-tract in all four species. Although determining the precise sequence downstream of a homopolymer longer than 20 nt is virtually impossible by Sanger sequencing due to polymerase slippage, the size of the homopolymer can be determined more reliably based on the peak heterogeneity downstream of the homopolymer^64^. Using this approach, the inferred length of the *y5-*m1 U-appendages was 48–50 nt.

### Phylogenetic inference

We used all 10 assigned mitochondrion-encoded protein sequences (Atp6, Cob, Cox1–3, and Nad1,4,5,7,8) from the four diplonemids and the corresponding homologs from other Discoba, notably *Hemistasia phaeocysticola* (Diplonemea); *Trypanosoma brucei* (Kinetoplastida); *Euglena gracilis* (Euglenida); *Acrasis kona*, *Naegleria gruberi*, and *Stachyamoeba lipophora* (Heterolobosea); *Andalucia godoyi*, *Reclinomonas americana* (ATCC 50394), and *Seculamonas ecuadoriensis* (Jakobida). Homologs from slowly evolving species were added as well, i.e., two from Malawimonada (*Malawimonas californiana* and *M. jakobiformis*) and four from Archaeplastida (*Porphyra purpurea* (Rhodophyta), *Prototheca wickerhamii*, *Klebsormidium flaccidum*, and *Marchantia polymorpha* (Chlorophyta)). Sequences were downloaded from the NCBI GenBank Protein database. Only one of the multiple allelic variants for each of the *H*. *phaeocysticola* genes was selected for the analysis. The sequences of Cox1, Cox2, and Nad1 from *M*. *jakobiformis*, including minor substitutions due to RNA editing, can be obtained upon request. Multiple sequence alignments (MSAs) of proteins were generated with Mams, a tool developed in-house (Lang B.F. and Rioux P., unpublished; available upon request), which produces, by employing Muscle v3.8.31^65^, a preliminary MSA for each protein class. These MSAs served to build HMMs, to which the protein sequences were aligned using hmmalign3 from the HMMER v3.0 package^55^. Alignment columns with posterior probabilities <1 were eliminated, and subsequently, protein sequences were concatenated for each species. The final MSA contained 19 taxa and 3,137 amino acid positions (the alignment is available from the authors upon request). Phylogenetic inferences were performed by a Bayesian approach using posterior probabilities as support values (PhyloBayes v3.2e^66^, MrBayes v3.2.6^67^) and by a Maximum Likelihood (ML) approach with bootstrapping (RAxML v8.2.11)^68^. Bayesian methods were executed in four independent chains and the first 25% cycles were discarded as burn-in. For Phylobayes, we chose the substitution model CAT-GTR, six discrete categories of gamma rate variation, and the -dc parameter to eliminate constant sites, and ~8,700 cycles corresponding to ~270,000 generations. For MrBayes, we chose the GTR model with six discrete categories of gamma rate variation, and 400,000 MCMC generations. For the ML tree, we used the substitution matrix LG for amino acid frequencies, eight distinct rate categories, a gamma model of rate heterogeneity with estimated parameters, the algorithm “rapid bootstrap analysis”, an optimization precision of 0.01 log likelihood units, and 100 distinct alternative runs on distinct starting trees for bootstrap support values.

### Analysis of structural domains of proteins

Sequences of Cox1, Nad4, and Nad5 proteins were aligned to their mitochondrial homologs with known three-dimensional structures from the Protein Data Bank (PDB)^69^ as described above. Structural alignments and models were inferred with Phyre2^70^ and visually examined in UCSF Chimera^71^.

## Electronic supplementary material

Supplementary Material
