# Supplementary material for: Keeping it complicated: Mitochondrial genome plasticity across diplonemids
Source: Sci Rep. 2017 Oct 26;7:14166. doi: 10.1038/s41598-017-14286-z (PMC5658414; doi:10.1038/s41598-017-14286-z)
Supplement: Supplementary file 1 — Supplementary Material [file 41598_2017_14286_MOESM1_ESM.pdf]

SUPPLEMENTARY MATERIAL

Keeping it complicated: Mitochondrial genome plasticity across diplonemids

Matus Valach [1,†], Sandrine Moreira [1,10,†], Steve Hoffmann [2], Peter F. Stadler [3,4,5,6,7,8,9], Gertraud Burger [1]

[1] Department of biochemistry and Robert-Cedergren Centre for Bioinformatics and Genomics, Université de Montréal, 2900 Edouard-Montpetit, Montreal H3T 1J4, QC, Canada
[2] Leipzig University, LIFE - Leipzig Research Center for Civilization Diseases, Haertelstrasse 16-18, Leipzig, D-04107, Germany
[3] Bioinformatics Group, Department Computer Science, and Interdisciplinary Center for Bioinformatics, University Leipzig, Härtelstrasse 16-18, D-04107 Leipzig, Germany
[4] German Centre for Integrative Biodiversity Research (iDiv) Halle-Jena-Leipzig, Competence Center for Scalable Data Services and Solutions, and Leipzig Research Center for Civilization Diseases, University Leipzig, D-04107 Leipzig, Germany
[5] Max Planck Institute for Mathematics in the Sciences, Inselstrasse 22, D-04103 Leipzig, Germany
[6] Fraunhofer Institute for Cell Therapy and Immunology, Perlickstrasse 1, D-04103 Leipzig, Germany
[7] Department of Theoretical Chemistry of the University of Vienna, Währingerstrasse 17, A-1090 Vienna, Austria
[8] Center for RNA in Technology and Health, University of Copenhagen, Grønnegårdsvej 3, 1870 Frederiksberg C, Denmark
[9] Santa Fe Institute, 1399 Hyde Park Road, Santa Fe, NM 87501, USA
[10] Present address: Department of Biochemistry and Molecular Biophysics, Columbia University, Hammer Health Science Center, 701 W 168th St, New York, NY 10031, USA

† These authors contributed equally to this work.

* Correspondence and requests for materials should be addressed to G.B. (email: gertraud.burger@umontreal.ca) or M.V. (email: matus.a.valach@gmail.com).

Supplementary Table S1. Catalog of all detected modules in the four diplonemids.

Gene	Module	Module ID [a]	5′ end [b]	3′ end [b]	Gene module size [c] [bp]	Transcript module size [d] [bp]
atp6	m1	*Da_atp6-m1*	curated	curated	162	162
		Dp_atp6-m1	curated	unambiguous	168	168
		Ds_atp6-m1	curated_homology	curated	165	165
		Re_atp6-m1	curated_homology	curated	156	156
	m2	*Da_atp6-m2*	curated	unambiguous	269	269
		Dp_atp6-m2	unambiguous	curated	269	269
		Ds_atp6-m2	curated	unambiguous	269	269
		Re_atp6-m2	curated	curated	269	269
	m3	*Da_atp6-m3*	unambiguous	ambiguous_U_-2	143	143
		Dp_atp6-m3	curated	ambiguous_U_-1	140	140
		Ds_atp6-m3	unambiguous	ambiguous_U_-1	140	143
		Re_atp6-m3	curated	curated_homology	142	142
cob	m1	*Da_cob-m1*	curated	curated	198	198
		Dp_cob-m1	curated	unambiguous	198	198
		Ds_cob-m1	curated_homology	curated	198	198
		Re_cob-m1	curated_homology	unambiguous	198	198
	m2	*Da_cob-m2*	curated	curated	154	154
		Dp_cob-m2	unambiguous	curated	154	154
		Ds_cob-m2	curated	curated	154	154
		Re_cob-m2	curated	unambiguous	154	154
	m3	*Da_cob-m3*	curated	curated	138	138
		Dp_cob-m3	curated	unambiguous	138	138
		Ds_cob-m3	curated	curated	138	138
		Re_cob-m3	curated	curated	138	138
	m4	*Da_cob-m4*	curated	curated	198	198
		Dp_cob-m4	unambiguous	unambiguous	198	198
		Ds_cob-m4	curated	curated	198	198
		Re_cob-m4	curated	unambiguous	198	198
	m5	*Da_cob-m5*	curated	unambiguous	279	279
		Dp_cob-m5	unambiguous	unambiguous	279	279
		Ds_cob-m5	curated	curated	279	279
		Re_cob-m5	curated	unambiguous	279	279
	m6	*Da_cob-m6*	unambiguous	unambiguous	123	126
		Dp_cob-m6	unambiguous	ambiguous_U_-1	123	126
		Ds_cob-m6	curated	ambiguous_U_-1	126	126
		Re_cob-m6	unambiguous	unambiguous	122	126
cox1	m1	*Da_cox1-m1*	curated	unambiguous	192	192
		Dp_cox1-m1	curated	curated	195	195
		Ds_cox1-m1	curated_homology	curated	189	189
		Re_cox1-m1	curated_homology	curated	189	189
	m2	*Da_cox1-m2*	curated	curated	124	124
		Dp_cox1-m2	curated	curated	124	124
		Ds_cox1-m2	curated	unambiguous	124	124
		Re_cox1-m2	curated	curated	124	124
	m3	*Da_cox1-m3*	curated	curated	263	263
		Dp_cox1-m3	curated	unambiguous	263	263
		Ds_cox1-m3	curated	unambiguous	263	263
		Re_cox1-m3	curated	curated	263	263
	m4	*Da_cox1-m4*	curated	unambiguous	220	226
		Dp_cox1-m4	unambiguous	unambiguous	220	226
		Ds_cox1-m4	unambiguous	unambiguous	220	226
		Re_cox1-m4	curated	unambiguous	220	226
	m5	*Da_cox1-m5*	unambiguous	curated	179	179
		Dp_cox1-m5	unambiguous	curated	179	179

Gene	Module	Module ID [a]	5′ end [b]	3′ end [b]	Gene module size [c] [bp]	Transcript module size [d] [bp]
		Ds_cox1-m5	curated	unambiguous	179	179
		Re_cox1-m5	unambiguous	curated	179	179
	m6	*Da_cox1-m6*	curated	unambiguous	169	169
		Dp_cox1-m6	curated	unambiguous	169	169
		Ds_cox1-m6	curated	unambiguous	169	169
		Re_cox1-m6	curated	curated	169	169
	m7	*Da_cox1-m7*	unambiguous	unambiguous	90	90
		Dp_cox1-m7	unambiguous	curated	90	90
		Ds_cox1-m7	curated	curated	90	90
		Re_cox1-m7	curated	unambiguous	90	90
	m8	*Da_cox1-m8*	unambiguous	curated	110	110
		Dp_cox1-m8	curated	curated	110	110
		Ds_cox1-m8	curated	unambiguous	110	110
		Re_cox1-m8	unambiguous	curated	110	110
	m9	*Da_cox1-m9*	curated	unambiguous	246	246
		Dp_cox1-m9	curated	unambiguous	249	250
		Ds_cox1-m9	unambiguous	ambiguous_UA_-4	237	237
		Re_cox1-m9	curated	curated	246	251
cox2	m1	*Da_cox2-m1*	curated	unambiguous	236	236
		Dp_cox2-m1	curated	curated	236	236
		Ds_cox2-m1	curated_homology	unambiguous	236	236
		Re_cox2-m1	curated_homology	unambiguous	236	236
	m2	*Da_cox2-m2*	unambiguous	curated	158	158
		Dp_cox2-m2	curated	curated	158	158
		Ds_cox2-m2	curated	unambiguous	158	158
		Re_cox2-m2	curated	unambiguous	158	158
	m3	*Da_cox2-m3*	curated	unambiguous	76	76
		Dp_cox2-m3	curated	unambiguous	76	76
		Ds_cox2-m3	unambiguous	curated	76	76
		Re_cox2-m3	unambiguous	curated	76	76
	m4	*Da_cox2-m4*	unambiguous	ambiguous_U_-2	126	128
		Dp_cox2-m4	unambiguous	unambiguous	125	128
		Ds_cox2-m4	curated	unambiguous	125	128
		Re_cox2-m4	curated	unambiguous	125	128
cox3	m1	*Da_cox3-m1*	curated	curated	338	338
		Dp_cox3-m1	curated	unambiguous	344	344
		Ds_cox3-m1	curated_homology	unambiguous	335	335
		Re_cox3-m1	curated_homology	curated	335	335
	m2	*Da_cox3-m2*	curated	unambiguous	266	266
		Dp_cox3-m2	unambiguous	curated	266	266
		Ds_cox3-m2	unambiguous	unambiguous	266	266
		Re_cox3-m2	curated	curated	266	266
	m3	*Da_cox3-m3*	unambiguous	ambiguous_U_-1	231	231
		Dp_cox3-m3	curated	unambiguous	230	231
		Ds_cox3-m3	unambiguous	ambiguous_U_-1	231	231
		Re_cox3-m3	curated	ambiguous_U_-1	223	225
nad1	m1	*Da_nad1-m1*	curated	unambiguous	189	189
		Dp_nad1-m1	curated	curated	189	189
		Ds_nad1-m1	curated_homology	curated	189	189
		Re_nad1-m1	curated_homology	curated	189	189
	m2	*Da_nad1-m2*	unambiguous	curated	114	114
		Dp_nad1-m2	curated	unambiguous	114	114
		Ds_nad1-m2	curated	unambiguous	114	114
		Re_nad1-m2	curated	unambiguous	114	114

Gene	Module	Module ID [a]	5′ end [b]	3′ end [b]	Gene module size [c] [bp]	Transcript module size [d] [bp]
	m3	*Da_nad1-m3*	curated	curated	166	166
		Dp_nad1-m3	unambiguous	curated	166	166
		Ds_nad1-m3	unambiguous	unambiguous	166	166
		Re_nad1-m3	unambiguous	curated	166	166
	m4	*Da_nad1-m4*	curated	unambiguous	233	233
		Dp_nad1-m4	curated	curated	233	233
		Ds_nad1-m4	curated	unambiguous	233	233
		Re_nad1-m4	curated	unambiguous	233	233
	m5	*Da_nad1-m5*	unambiguous	unambiguous	205	220
		Dp_nad1-m5	curated	unambiguous	204	220
		Ds_nad1-m5	unambiguous	unambiguous	200	217
		Re_nad1-m5	curated	ambiguous_U_-2	200	220
nad4	m1	*Da_nad4-m1*	curated_homology	unambiguous	247	247
		Dp_nad4-m1	curated	curated	247	247
		Ds_nad4-m1	curated_homology	unambiguous	247	247
		Re_nad4-m1	curated_homology	curated	247	247
	m2	*Da_nad4-m2*	unambiguous	curated	225	225
		Dp_nad4-m2	curated	curated	225	225
		Ds_nad4-m2	unambiguous	unambiguous	225	225
		Re_nad4-m2	curated	unambiguous	225	225
	m3	*Da_nad4-m3*	curated	curated	171	171
		Dp_nad4-m3	curated	unambiguous	168	168
		Ds_nad4-m3	unambiguous	unambiguous	168	168
		Re_nad4-m3	curated	unambiguous	168	168
	m4	*Da_nad4-m4*	curated	unambiguous	134	136
		Dp_nad4-m4	unambiguous	unambiguous	134	136
		Ds_nad4-m4	curated	curated	131	133
		Re_nad4-m4	unambiguous	curated	134	136
	m5	*Da_nad4-m5*	unambiguous	unambiguous	74	74
		Dp_nad4-m5	unambiguous	unambiguous	74	74
		Ds_nad4-m5	unambiguous	unambiguous	74	74
		Re_nad4-m5	curated	curated	74	74
	m6	*Da_nad4-m6*	unambiguous	unambiguous	165	165
		Dp_nad4-m6	unambiguous	unambiguous	165	165
		Ds_nad4-m6	curated	unambiguous	165	165
		Re_nad4-m6	curated	unambiguous	165	165
	m7	*Da_nad4-m7*	unambiguous	curated	120	120
		Dp_nad4-m7	unambiguous	unambiguous	120	120
		Ds_nad4-m7	unambiguous	curated	120	120
		Re_nad4-m7	unambiguous	unambiguous	120	120
	m8	*Da_nad4-m8*	curated	curated	164	171
		Dp_nad4-m8	unambiguous	unambiguous	170	172
		Ds_nad4-m8	curated	ambiguous_U_-1	174	174
		Re_nad4-m8	curated	curated	143	149
nad5	m1	*Da_nad5-m1*	curated_homology	unambiguous	288	288
		Dp_nad5-m1	curated	curated	291	291
		Ds_nad5-m1	curated_homology	curated	294	294
		Re_nad5-m1	curated_homology	curated	288	288
	m2	*Da_nad5-m2*	unambiguous	curated	218	218
		Dp_nad5-m2	curated	unambiguous	218	218
		Ds_nad5-m2	curated	curated	218	218
		Re_nad5-m2	curated	unambiguous	218	218
	m3	*Da_nad5-m3*	curated	unambiguous	57	57
		Dp_nad5-m3	unambiguous	unambiguous	60	60

Gene	Module	Module ID [a]	5′ end [b]	3′ end [b]	Gene module size [c] [bp]	Transcript module size [d] [bp]
		Ds_nad5-m3	curated	curated	66	66
		Re_nad5-m3	curated	curated	57	57
	m4	*Da_nad5-m4*	unambiguous	curated	186	186
		Dp_nad5-m4	unambiguous	unambiguous	183	183
		Ds_nad5-m4	curated	unambiguous	183	183
		Re_nad5-m4	curated	unambiguous	183	183
	m5	*Da_nad5-m5*	curated	unambiguous	136	136
		Dp_nad5-m5	unambiguous	unambiguous	136	136
		Ds_nad5-m5	unambiguous	unambiguous	136	136
		Re_nad5-m5	curated	unambiguous	136	136
	m6	*Da_nad5-m6*	unambiguous	unambiguous	122	122
		Dp_nad5-m6	unambiguous	curated	122	122
		Ds_nad5-m6	unambiguous	curated	122	122
		Re_nad5-m6	unambiguous	unambiguous	122	122
	m7	*Da_nad5-m7*	unambiguous	unambiguous	188	195
		Dp_nad5-m7	curated	unambiguous	201	201
		Ds_nad5-m7	curated	unambiguous	194	201
		Re_nad5-m7	curated	curated	192	192
	m8	*Da_nad5-m8*	unambiguous	curated	84	84
		Dp_nad5-m8	unambiguous	unambiguous	90	90
		Ds_nad5-m8	curated	unambiguous	81	81
		Re_nad5-m8	curated	curated	87	87
	m9	*Da_nad5-m9*	curated	curated	182	182
		Dp_nad5-m9	unambiguous	curated	191	191
		Ds_nad5-m9	curated	unambiguous	188	188
		Re_nad5-m9	curated	curated	179	179
	m10	*Da_nad5-m10*	curated	curated	116	116
		Dp_nad5-m10	curated	unambiguous	113	113
		Ds_nad5-m10	unambiguous	unambiguous	125	125
		Re_nad5-m10	curated	curated	104	110
	m11	*Da_nad5-m11*	curated	ambiguous_U_-1	48	73
		Dp_nad5-m11	unambiguous	ambiguous_U_-1	73	73
		Ds_nad5-m11	curated	curated	75	76
		Re_nad5-m11	curated	unambiguous	47	73
nad7	m1	*Da_nad7-m1*	curated	unambiguous	173	173
		Dp_nad7-m1	curated	curated	173	173
		Ds_nad7-m1	curated_homology	curated	173	173
		Re_nad7-m1	curated_homology	curated	173	173
	m2	*Da_nad7-m2*	unambiguous	unambiguous	75	75
		Dp_nad7-m2	curated	unambiguous	75	75
		Ds_nad7-m2	curated	curated	75	75
		Re_nad7-m2	curated	curated	75	75
	m3	*Da_nad7-m3*	unambiguous	unambiguous	133	133
		Dp_nad7-m3	unambiguous	unambiguous	133	133
		Ds_nad7-m3	curated	unambiguous	133	133
		Re_nad7-m3	curated	curated	133	133
	m4	*Da_nad7-m4*	unambiguous	unambiguous	64	64
		Dp_nad7-m4	unambiguous	unambiguous	64	64
		Ds_nad7-m4	unambiguous	curated	64	64
		Re_nad7-m4	curated	curated	64	64
	m5	*Da_nad7-m5*	unambiguous	unambiguous	192	192
		Dp_nad7-m5	unambiguous	curated	192	192
		Ds_nad7-m5	curated	unambiguous	186	186
		Re_nad7-m5	curated	unambiguous	189	189

Gene	Module	Module ID [a]	5′ end [b]	3′ end [b]	Gene module size [c] [bp]	Transcript module size [d] [bp]
	m6	*Da_nad7-m6*	unambiguous	curated	66	66
		Dp_nad7-m6	curated	unambiguous	66	66
		Ds_nad7-m6	unambiguous	unambiguous	66	66
		Re_nad7-m6	curated	curated	66	66
	m7	*Da_nad7-m7*	curated	curated	169	169
		Dp_nad7-m7	unambiguous	unambiguous	169	169
		Ds_nad7-m7	unambiguous	unambiguous	157	157
		Re_nad7-m7	curated	unambiguous	166	166
	m8	*Da_nad7-m8*	curated	curated	176	176
		Dp_nad7-m8	unambiguous	curated	182	182
		Ds_nad7-m8	unambiguous	unambiguous	179	179
		Re_nad7-m8	unambiguous	curated	185	185
	m9	*Da_nad7-m9*	curated	ambiguous_U_-1	78	78
		Dp_nad7-m9	curated	ambiguous_UA_-2	79	79
		Ds_nad7-m9	curated	ambiguous_U_-1	78	78
		Re_nad7-m9	curated	ambiguous_UA_-2	79	79
nad8	m1	*Da_nad8-m1*	curated_homology	unambiguous	144	144
		Dp_nad8-m1	curated	unambiguous	144	144
		Ds_nad8-m1	curated_homology	unambiguous	150	150
		Re_nad8-m1	curated_homology	unambiguous	144	144
	m2	*Da_nad8-m2*	unambiguous	curated	153	153
		Dp_nad8-m2	unambiguous	curated	153	153
		Ds_nad8-m2	unambiguous	unambiguous	153	153
		Re_nad8-m2	curated	unambiguous	150	150
	m3	*Da_nad8-m3*	curated	curated	78	85
		Dp_nad8-m3	curated	unambiguous	72	84
		Ds_nad8-m3	unambiguous	curated	75	76
		Re_nad8-m3	unambiguous	curated	81	89
rnl	m1	*Da_rnl-m1*	curated	unambiguous	542	568
		Dp_rnl-m1	curated	curated	534	560
		Ds_rnl-m1	curated_homology	unambiguous	521	546
		Re_rnl-m1	curated_homology	ambiguous_U_-1	525	555
	m2	*Da_rnl-m2*	curated	curated	352	352
		Dp_rnl-m2	curated	curated	352	352
		Ds_rnl-m2	ambiguous_U_+1	curated	339	339
		Re_rnl-m2	unambiguous	curated	340	340
rns	m1	*Da_rns-m1*	curated	curated	372	381
		Dp_rns-m1	curated	curated	366	374
		Ds_rns-m1	curated	ambiguous_U_-1	386	391
		Re_rns-m1	curated_homology	unambiguous	363	372
y1	m1	*Da_y1-m1*	curated_homology	curated	241	241
		Dp_y1-m1	curated	unambiguous	241	241
		Ds_y1-m1	curated_homology	unambiguous	244	244
		Re_y1-m1	curated_homology	unambiguous	241	241
	m2	*Da_y1-m2*	curated	curated	77	97
		Dp_y1-m2	unambiguous	ambiguous_U_-1	101	105
		Ds_y1-m2	unambiguous	ambiguous_U_-3	100	102
		Re_y1-m2	curated	curated	83	94
y2	m1	*Da_y2-m1*	curated_homology	curated	241	241
		Dp_y2-m1	curated_homology	unambiguous	235	235
		Ds_y2-m1	curated_homology	curated	244	244
		Re_y2-m1	curated_homology	curated	242	242
	m2	*Da_y2-m2*	curated	curated	97	97
		Dp_y2-m2	unambiguous	curated	94	94

Gene	Module	Module ID [a]	5′ end [b]	3′ end [b]	Gene module size [c] [bp]	Transcript module size [d] [bp]
		Ds_y2-m2	curated	unambiguous	73	73
		Re_y2-m2	curated	unambiguous	78	78
	m3	*Da_y2-m3*	curated	unambiguous	93	111
		Dp_y2-m3	curated	ambiguous_U_-1	97	115
		Ds_y2-m3	curated	unambiguous	96	115
		Re_y2-m3	unambiguous	ambiguous_U_-1	97	115
	m4	*Da_y2-m4*	curated	ambiguous_U_-1	68	68
		Dp_y2-m4	unambiguous	unambiguous	53	64
		Ds_y2-m4	curated	unambiguous	62	67
		Re_y2-m4	unambiguous	curated	58	64
y3	m1	*Da_y3-m1*	curated_homology	unambiguous	179	179
		Dp_y3-m1	curated	unambiguous	194	194
		Re_y3-m1	curated_homology	curated	170	170
	m1m2	*Ds_y3-m1m2*	curated_homology	unambiguous	285	285
	m2	*Da_y3-m2*	unambiguous	unambiguous	109	109
		Dp_y3-m2	unambiguous	curated	109	109
		Re_y3-m2	curated	unambiguous	106	106
		Da_y3-m3	unambiguous	unambiguous	141	168
	m3	*Dp_y3-m3*	curated	unambiguous	141	168
		Ds_y3-m3	unambiguous	unambiguous	140	168
		Re_y3-m3	unambiguous	ambiguous_U_-1	144	171
	m4	*Da_y3-m4*	curated	ambiguous_U_-1	360	375
		Dp_y3-m4	unambiguous	ambiguous_U_-1	368	384
		Ds_y3-m4	unambiguous	curated	368	384
		Re_y3-m4	unambiguous	ambiguous_U_-1	367	381
	m5	*Da_y3-m5*	curated	unambiguous	133	136
		Dp_y3-m5	unambiguous	unambiguous	126	127
		Ds_y3-m5	unambiguous	ambiguous_U_-1	129	136
		Re_y3-m5	unambiguous	ambiguous_U_-1	128	130
y5	m1	*Da_y5-m1*	curated_homology	ambiguous_U_-1	186	236
		Dp_y5-m1	curated	unambiguous	195	245
		Ds_y5-m1	curated_homology	ambiguous_U_-1	198	248
		Re_y5-m1	curated_homology	unambiguous	194	242
	m2	*Da_y5-m2*	unambiguous	curated	109	109
		Dp_y5-m2	unambiguous	unambiguous	109	109
		Ds_y5-m2	curated	unambiguous	121	121
		Re_y5-m2	ambiguous_U_+1	unambiguous	109	109
	m3	*Da_y5-m3*	curated	curated	39	55
		Dp_y5-m3	unambiguous	ambiguous_A_-1	57	62
		Ds_y5-m3	unambiguous	curated	36	56
		Re_y5-m3	unambiguous	ambiguous_U_-1	56	58
y6	m1	*Da_y6-m1*	curated	unambiguous	192	192
		Dp_y6-m1	curated	unambiguous	192	192
		Ds_y6-m1	curated_homology	curated	192	192
		Re_y6-m1	curated_homology	unambiguous	188	188
	m2	*Da_y6-m2*	unambiguous	curated	45	45
		Dp_y6-m2	unambiguous	unambiguous	43	49
		Ds_y6-m2	curated	curated_homology	45	45
		Re_y6-m2	curated	curated_homology	46	46

[a] Module ID consists of species abbreviation, gene name, and module number. Da, *D. ambulator*; Dp, *D. papillatum*; Ds, *D.* sp. 2; Re, *R. euleeides*.
[b] Terminal nucleotide of a module was inferred: i) from sequence alone ('unambiguous'); ii) experimentally ('curated'); iii) based on the homology to other species ('curated_homology'). Remaining, ambiguous termini

(‘ambiguous’) are labelled according to the nucleotide residue, whose origin (genome-encoded or post-transcriptionally added) could not be determined (U, uridine; A, adenosine). The number and its sign indicate the maximum distance and orientation, respectively, of the shift. For example, in the case of *Da_atp6-m3*, ‘ambiguous_U_-2’ means that the last two Us could be either genome-encoded, or appended, with the indicated size (e.g. 143 bp) corresponding to the default situation where the two Us are genome-encoded; when the two Us are appended by RNA editing, the true size of the module would be 141 bp (=143−2).

[c] Size of the module’s coding portion (excluding 5′ UTR and 3′ UTR).

[d] Size of the mature module transcript, including the appended Us (all modules) and 3′ UTR (only last modules). Adenosine residues added by polyadenylation and 5′ UTR of the first modules are not counted. UTR length has been precisely determined for all genes from *D. papillatum* except for *y2*, but only for a few genes from the other species (for details, compare ‘curated’ and ‘curated_homology’ in the column ‘5′ end’ of m1).

Supplementary Table S2. Characterization of diplonemid mitochondrial chromosomes.

Species	Chromosome name [a]	Module(s)	Cassette subclass [b]	Mean coverage [c]	GenBank Acc. Nr.
D. ambulator	A01 (4,875)	*cox1-m9*	a1	10150.2	JF698651, MF436754
(*Da_*)	A02	*rnl-m2*	a2	10401.8	MF436783
	A02	*nad1-m5, y2-m3*	a2*	11061.4	MF436783
	A03	*cox1-m1*	a1	4727.7	JF698675, MF436748
	A04	*cox1-m3*	a2	8900.1	JF698677, MF436750
	A05	*rns-m1*	a2	28480.9	MF436784
	A06	*rnl-m1*	a2	25131.6	MF436782
	A07	*y6-m1*	a1	1819.7	MF436789
	A08	*y3-m4*	a1	11564.6	MF436787
	A09	*y1-m1*	a1	18087.5	MF436785
	A10	*cob-m2, y2-m4*	a1	14939.2	MF436745
	A11	*nad7-m3, y2-m2, y5-m3*	a1	5815.7	MF436773
	A12	*cox2-m4*	a2	2875.7	MF436758
	A13	*nad4-m8*	a2	587.7	MF436765
	A14	*nad7-m4*	a1	10807.6	MF436774
	B01 (4,959)	*cox1-m4*	b	5631.0	JF698653, MF436751
	B02	*cox2-m1, cox1-m8*	b	11250.9	JF698680, MF436755
	B03	*nad5-m9*	b	15026.8	MF436769
	B04	*cob-m1*	b	6783.1	MF436744
	B05	*nad5-m1*	b	1713.4	MF436766
	B06	*atp6-m2*	b	6966.6	MF436742
	B07	*cox1-m7*	b	3556.2	JF698679, MF436753
	C01	*nad8-m1, nad4-m2, cox3-m2*	c1	2681.6	MF436779
	C02	*cox3-m3*	c1	4234.8	MF436759
	C03	*nad8-m3, nad5-m4, atp6-m1, y2-m1*	c1	2978.1	MF436781
	C04	*nad5-m3, nad5-m10*	c1	2243.6	MF436767
	C04	*nad5-m11*	c2	2488.4	MF436770
	C05	*nad7-m2, nad5-m2, nad4-m5, cox1-m6, y1-m2*	c1	4973.1	JF698678, MF436772
	C05	*X5*	c2	3263.8	MF436794
	C06	*nad8-m2, nad5-m7*	c1	2130.0	MF436780
	C07	*y3-m5*	c1	4891.0	MF436788
	C07	*nad1-m2*	c2	4544.3	MF436760
	C08	*y3-m1*	c1	2981.2	MF436786
	C09	*cox2-m2, y3-m2*	c1	2334.7	MF436756
	C10	*nad7-m7*	c1	5777.7	MF436776
	C11	*nad7-m8*	c1	1744.4	MF436777
	C12	*nad4-m4, y5-m2, y6-m2*	c1	2975.4	MF436764
	C12	*atp6-m3*	c2	2637.2	MF436743
	C13	*nad1-m4*	c1	4983.7	MF436762
	C14	*cob-m4, cob-m5*	c1	5246.4	MF436747
	C*	*cob-m3*	c2	2446.7	MF436746
	C*	*cox1-m2*	c2	2709.1	JF698676, MF436749
	C*	*cox2-m3*	c2	1971.1	MF436757
	C*	*nad5-m6*	c2	5089.2	MF436768
	C*	*nad7-m1*	c2	4801.5	MF436771

Species	Chromosome name [a]	Module(s)	Cassette subclass [b]	Mean coverage [c]	GenBank Acc. Nr.
	C*	*X1*	c2	3709.9	MF436790
	C*	*X2*	c2	4296.8	MF436791
	C*	*X3*	c2	1649.6	MF436792
	C*	*X4*	c2	5072.4	MF436793
	C*	*X6*	c2	726.4	MF436795
	U01	*nad1-m3, nad7-m6*		6003.7	MF436761
	U01	*nad5-m8, nad5-m5, nad4-m7, nad1-m1, cox3-m1, cob-m6*		6387.3	MF436761
	U02 (4,593)	*cox1-m5, nad4-m3*		12456.2	JF698652, MF436752
	U02 (4,593)	*y3-m3, y5-m1*		11874.2	JF698652, MF436752
	U03	*nad7-m9, nad4-m6*		5542.5	MF436778
	U04	*nad7-m5*		4818.0	MF436775
	U05	*nad4-m1*		1973.7	MF436763
D. sp. 2	A01 (5,184)	*cox1-m9, nad5-m3*	a4	479.5	JF698654, MF436810
(*Ds_*)	A02 (5,163)	*cox1-m5*	a3	347.1	JF698655, MF436808
	A03	*nad7-m2*	a4*	1489.1	MF436839
	A04	*cox3-m2*	a1*	2408.6	MF436816
	A05	*y6-m2*	a2*	4657.6	MF436864
	A06	*nad7-m1*	a1*	1503	MF436838
	A07	*nad5-m7*	a2	2627.2	MF436834
	A08	*cob-m1*	a3	8631.3	MF436799
	A09	*nad4-m2*	a1	3705.6	MF436824
	A10	*nad4-m1*	a1	768.7	MF436823
	A11	*nad5-m5*	a2	2047.5	MF436832
	A12	*nad1-m4*	a2	2697.4	MF436821
	A13	*cob-m5*	a1	1055.9	MF436803
	A14	*y1-m1*	a1	155.4	MF436854
	A15	*cob-m2*	a1	831.8	MF436800
	A16	*atp6-m3*	a4*	7254.9	MF436798
	A17	*nad1-m1*	a1	392.1	MF436818
	A18	*nad1-m5*	a1	1094.8	MF436822
	A19	*nad7-m8*	a3	988.8	MF436843
	A20	*cox2-m1*	a1	1139.7	MF436811
	A21	*nad7-m4*	a4*	1941.4	MF436840
	A22	*nad8-m2*	a1*	414.2	MF436846
	A23	*nad5-m4*	a1	712.8	MF436831
	A24	*y2-m1*	a1	7811.6	MF436856
	A25	*cox3-m3*	a1	464.8	MF436817
	A26	*cox1-m1*	a2	202.4	JF698658, MF436804
	A27	*nad8-m1*	a3	1211.1	MF436845
	A28	*nad4-m8*	a1	1396.7	MF436828
	A29	*nad5-m9*	a1	7425.5	MF436836
	A30	*nad1-m3*	a1	1124.1	MF436820
	A31	*nad4-m6*	a2	8737.7	MF436827
	A32	*y2-m4*	a1	19898.9	MF436858
	A33	*cox2-m3, cox1-m7*	a1	4163.9	JF698662, MF436813
	A34	*cob-m4*	a4	332	MF436802
	A35	*X3*	a4*	1273.1	MF436852
	A36	*y5-m1*	a1	2060	MF436861
	A37	*nad7-m7*	a3	755.6	MF436842
	A38	*nad1-m2*	a1	619.2	MF436819
	A39	*y6-m1*	a4	1684.6	MF436863

Species	Chromosome name [a]	Module(s)	Cassette subclass [b]	Mean coverage [c]	GenBank Acc. Nr.
	A40	*nad8-m3*	a2	8364.5	MF436847
	A41	*nad5-m8*	a4	2288.7	MF436835
	A42	*atp6-m1*	a3	10697.6	MF436796
	A43	*X4*	a4*	1140.2	MF436853
	B01	*y3-m4*	b	7715.6	MF436860
	B02	*nad7-m5*	b	9931.5	MF436841
	B03	*cox3-m1*	b	7185.3	MF436815
	B04	*cox1-m3*	b	9401.9	JF698660, MF436806
	B05	*cox1-m6*	b	10342.8	JF698661, MF436809
	B06	*cox2-m2*	b	10024.3	MF436812
	B07	*nad4-m5, cox1-m8*	b	7159.1	JF698663, MF436826
	B08	*nad5-m2*	b	6114.4	MF436830
	B09	*y3-m3, y5-m3*	b	8785	MF436859
	B10	*nad5-m10*	b	6286.9	MF436837
	B11	*y1-m2*	b	6310.6	MF436855
	B12	*nad7-m9*	b	8868.9	MF436844
	C01	*cob-m3*	c	4838.9	MF436801
	C02	*cox1-m2*	c	3001.9	JF698659, MF436805
	C03	*nad4-m3*	c	5710.9	MF436825
	C04	*X2*	c	1202.3	MF436851
	C05	*X1*	c	311.0	MF436850
	D01	*rns-m1*	d	3076.4	MF436849
	D02	*cox2-m4*	d	4288.2	MF436814
	D03	*rnl-m1*	d	4857.4	MF436848
	U01	*cox1-m4, nad4-m4, y3-m1m2*		1081.3	JF698656, MF436807
	U01	*rnl-m2*		1132.5	JF698656, MF436807
	U02 (7,130)	*y2-m2, cob-m6*		838.6	MF436857
	U03 (6,059)	*y5-m2*		731.8	MF436862
	U04 (5,770)	*nad5-m1*		911.7	MF436829
	U05 (5,388)	*atp6-m2, nad7-m6, y2-m3*		1105.6	MF436797
	U05 (5,388)	*y3-m5*		1048.8	MF436797
	U06	*nad5-m6, nad7-m3, nad5-m11*		2129.8	MF436833
	U06	*nad4-m7*		1872.6	MF436833
R. euleeides (*Re*)	A01	*rnl-m1*	a3*	8091.7	MF436917
	A02	*nad5-m1*	a3	4102.5	MF436897
	A03	*nad5-m9*	a2	5043.4	MF436904
	A04	*cox1-m2*	a1	1115.8	JF698670, MF436874
	A05	*nad5-m11*	a3	1898.2	MF436906
	A06	*cob-m3*	a2	577.8	MF436869
	A07	*nad1-m2*	a2	6472.0	MF436887
	A08	*rns-m1*	a3	8738.5	MF436919
	A09	*y5-m1*	a1	3839.8	MF436928
	A10	*y3-m4*	a3	262.1	MF436926
	A11	*nad1-m3*	a3	11530.7	MF436888
	A12	*cox2-m2*	a1	932.6	MF436881
	A13	*cox3-m1*	a3	1895.2	MF436883
	A14	*nad8-m2*	a1	5529.7	MF436915
	A15	*nad7-m1*	a1	899.9	MF436907
	A16	*y3-m3*	a3	4073.1	MF436925
	A17	*y3-m1*	a3	4270.5	MF436924
	A18	*nad5-m8, nad4-m2*	a3	2767.0	MF436903

Species	Chromosome name [a]	Module(s)	Cassette subclass [b]	Mean coverage [c]	GenBank Acc. Nr.
	A19	*nad7-m8*	a1	21966.9	MF436913
	A20	*cob-m4*	a1	1269.4	MF436870
	A21	*y2-m3, y2-m4*	a1	355.1	MF436923
	A22	*nad5-m6*	a1	16173.2	MF436901
	A23	*y3-m5*	a1	1041.5	MF436927
	A24	*nad1-m5*	a3	9795.8	MF436890
	A25	*cob-m5, y5-m3*	a3	978.2	MF436871
	A26	*y5-m2*	a3	717.6	MF436929
	A27	*cob-m6*	a3	555.6	MF436872
	A28	*atp6-m3*	a2	675.7	MF436866
	A29	*cox3-m2*	a3	536.4	MF436884
	A30	*cox1-m3*	a3	542.8	JF698671, MF436875
	A31	*cox1-m4*	a1	277.7	JF698667, MF436876
	A32	*nad4-m1, y1-m2*	a3	5322.2	MF436891
	A33	*cox1-m9*	a3	868.0	JF698664, MF436879
	A34	*y1-m1*	a3	4980.2	MF436920
	A35	*cox2-m1*	a3	1158.4	MF436880
	A36	*nad1-m4*	a3	1659.6	MF436889
	A37	*y2-m1*	a3	772.8	MF436921
	A38	*cox3-m3*	a3	2539.1	MF436885
	A39	*nad7-m5*	a1	497.8	MF436910
	A40	*nad5-m2*	a3	2232.5	MF436898
	A41	*nad7-m7*	a3	308.9	MF436912
	A42	*cox1-m1*	a3	4975.5	JF698669, MF436873
	A43	*y6-m1*	a3	1051.9	MF436930
	A44	*nad5-m7*	a3	755.8	MF436902
	A45	*nad5-m4*	a3	4132.5	MF436900
	A46	*cox1-m5*	a3	7731.6	JF698666, MF436877
	A47	*nad4-m8*	a3	1347.7	MF436896
	A48	*cob-m1*	a3	6201.6	MF436867
	A49	*nad4-m4*	a1	191.9	MF436893
	A50	*nad4-m3*	a3	1830.9	MF436892
	A51	*cob-m2*	a3	578.9	MF436868
	A52	*nad7-m6*	a3	1418.1	MF436911
	B01	*nad8-m1, nad5-m5, cox2-m3*	b	1515.1	MF436914
	B02	*nad5-m3, nad4-m5, y6-m2, atp6-m1*	b*	1305.8	MF436899
	B03	*nad8-m3, nad7-m4*	b	1290.1	MF436916
	B04	*nad4-m6*	b	446.0	MF436894
	B05	*cox1-m7*	b	1340.3	JF698673, MF436878
	B06	*nad5-m10*	b	813.0	MF436905
	B07	*nad7-m2*	b	841.6	MF436908
	C01	*nad4-m7*	c	15616.5	MF436895
	C02	*atp6-m2*	c*	4222.5	MF436865
	C03	*rnl-m2*	c	3165.0	MF436918
	C04	*y2-m2, y3-m2*	c	1337.0	MF436922
	D01	*nad1-m1*	d	1246.9	MF436886
	D02	*nad7-m3, cox1-m8*	d	1259.2	JF698674, MF436909
	U01	*cox2-m4, cox1-m6, nad7-m9*		1292.3	JF698672, MF436882
D. papillatum (*Dp_*)	A01	*atp6-m1*	a	2939.2	KU356490
	A02	*atp6-m2*	a	686.4	KU356491

Species	Chromosome name [a]	Module(s)	Cassette subclass [b]	Mean coverage [c]	GenBank Acc. Nr.
	A03	*atp6-m3*	a	8730.5	KU356492
	A04	*cob-m1*	a	724.4	KU356493
	A05	*cob-m2*	a	5114.5	KU356494
	A06	*cob-m3*	a	6277.4	KU356495
	A07	*cob-m4*	a	2562.3	KU356496
	A08	*cob-m5*	a	2276.0	KU356497
	A09	*cox1-m2*	a	4928.6	KU356500
	A10	*cox1-m3*	a	21655.6	KU356501
	A11	*cox1-m5*	a	1790.7	KU356503
	A12	*cox1-m6*	a	1202.9	KU356504
	A13	*cox1-m7*	a	549.2	KU356505
	A14	*cox1-m8*	a	2060.5	KU356506
	A15 (5,802; 5,852) †	*cox1-m9*	a	10148.0	KU356507, HQ288823, EU123536
	A16	*cox2-m1*	a	9229.5	KU356508
	A17	*cox2-m3*	a	994.3	KU356510
	A18	*cox2-m4*	a	723.9	KU356511
	A19	*cox3-m1*	a	5418.4	KU356512
	A20	*cox3-m2*	a	2359.6	KU356513
	A21	*cox3-m3*	a	7666.8	KU356514
	A22	*nad1-m1*	a	7552.8	KU356515
	A23	*nad1-m2*	a	12429.3	KU356516
	A24	*nad1-m3*	a	4863.8	KU356517
	A25	*nad1-m4*	a	4640.3	KU356518
	A26	*nad1-m5*	a	7701.2	KU356519
	A27	*nad4-m1*	a	14167.1	KU356520
	A28	*nad4-m3*	a	5146.9	KU356522
	A29	*nad4-m4*	a	4945.9	KU356523
	A30	*nad4-m6*	a	8085.8	KU356525
	A31	*nad4-m8*	a	4331.2	KU356527
	A32	*nad5-m10, y5-m3*	a	4856.4	KU356537
	A33	*nad5-m11, y2-m2*	a	8446.4	KU356538
	A34	*nad5-m2*	a	6914.4	KU356529
	A35	*nad5-m3*	a	1445.1	KU356530
	A36	*nad5-m4*	a	1030.1	KU356531
	A37	*nad5-m5*	a	868.2	KU356532
	A38	*nad5-m6*	a	1514.6	KU356533
	A39	*nad5-m7*	a	4044.9	KU356534
	A40	*nad5-m8*	a	8618.5	KU356535
	A41	*nad5-m9*	a	4039.5	KU356536
	A42	*nad7-m1*	a	2336.1	KU356539
	A43	*nad7-m2*	a	7340.5	KU356540
	A44	*nad7-m3*	a	1773.4	KU356541
	A45	*nad7-m5*	a	8342.8	KU356543
	A46 (5,794)	*nad7-m6, y2-m3*	a	5970.0	KU356544, HQ288824
	A47 (5,763)	*nad7-m8*	a	826.1	KU356546, JQ302962
	A48	*nad7-m9*	a	843.1	KU356547
	A49	*nad8-m1*	a	5728.1	KU356548
	A50	*nad8-m3*	a	8262.4	KU356550
	A51	*y1-m1*	a	4954.4	KU356554
	A52	*y1-m2*	a	22343.9	KU356555
	A53	*y2-m4*	a	2798.8	KU356557
	A54	*y3-m1*	a	6392.6	KU356558
	A55	*y3-m5*	a	10054.6	KU356562
	A56	*y4-m1*	a	2222.5	KU356563

**Species**	**Chromosome name [a]**	**Module(s)**	**Cassette subclass [b]**	**Mean coverage [c]**	**GenBank Acc. Nr.**
	A57	*y4-m2*	a	2322.4	KU356564
	A58	*y5-m1*	a	8032.7	KU356565
	A59	*y6-m2*	a	1146.5	KU356568
	A60	*X12-m*	a	1219.2	KU356569
	A61	*X18-m*	a	4213.1	KU356570
	B01	*cob-m6*	b	3069.1	KU356498
	B02	*cox1-m1*	b*	2977.4	KU356499
	B03 (7,182)	*cox1-m4*	b	10329.8	KU356502, EU123537
	B04	*cox2-m2*	b	1573.9	KU356509
	B05	*nad4-m2*	b*	6250.0	KU356521
	B06	*nad4-m5*	b*	1649.1	KU356524
	B07	*nad4-m7*	b	6485.5	KU356526
	B08	*nad5-m1*	b	1582.5	KU356528
	B09	*nad7-m4*	b*	5688.5	KU356542
	B10	*nad7-m7*	b	14312.6	KU356545
	B11	*nad8-m2*	b	1878.6	KU356549
	B12	*rnl-m1*	b*	29748.1	KU356551
	B13	*rnl-m2*	b	10837.6	KU356552
	B14	*rns-m1*	b	8776.9	KU356553
	B15	*y2-m1*	b*	953.5	KU356556
	B16	*y3-m2*	b	689.4	KU356559
	B17	*y3-m3*	b	17358.5	KU356560
	B18	*y3-m4*	b	597.6	KU356561
	B19	*y5-m2*	b	1318.0	KU356566
	B20	*y6-m1*	b	5169.8	KU356567

[a] Chromosome name consists of a letter referring to the class (A-D, U) and an ordinal number. For a chromosome that was completely sequenced the length is indicated in parentheses. The c2 cassettes labeled with an asterisk most likely associate with one of the c1 cassettes to form a bi-cassette C-class chromosome (i.e., *Da*_C01-C03, C06, C08-C11, C13-C14), but the exact partners have not been determined. Note that for chromosomes with two cassettes (or module arrays), the chromosome name is listed twice to separately show modules belonging to each cassette (or array). The dagger symbol indicates the chromosome, whose two alleles were completely sequenced (*Dp*_A15).
[b] Cassettes are categorized according to their class (A-D, U) with a lowercase letter referring to the class and the number referring to a subclass, which is based on the variant cassette-framing motifs. The asterisk indicates a derived cassette (e.g., identity below 90% compared to the usual recurrent framing motif for that class either upstream, or downstream of the cassette).
[c] Mean coverage by sequencing reads was calculated for the entire length of a cassette or for a module array plus 50 bp upstream and downstream (see Methods for details). *D. papillatum* data were taken from [1].

Supplementary Table S3. Editing sites in the mitochondrial genes of *D/R* diplonemids.

Gene	Species	Nr. of U-appendage sites [a]	U-tract length [nt]	Nr. of substitution clusters (Total edits)	Nr. of A-to-I sites	Nr. of C-to-U sites
atp6	*D. ambulator*	0	0			
	D. papillatum	0	0			
	D. sp. 2	1	3			
	R. euleeides	n.d.	n.d.			
cob	*D. ambulator*	1	3			
	D. papillatum	1	3			
	D. sp. 2	0	0			
	R. euleeides	1	4			
cox1	*D. ambulator*	1	6			
	D. papillatum	1	6			
	D. sp. 2	1	6			
	R. euleeides	1	6			
cox2	*D. ambulator*	1	2			
	D. papillatum	1	3			
	D. sp. 2	1	3			
	R. euleeides	1	3			
cox3	*D. ambulator*	0	0			
	D. papillatum	1	1			
	D. sp. 2	0	0			
	R. euleeides	1	2			
nad1	*D. ambulator*	1	15			
	D. papillatum	1	16			
	D. sp. 2	1	17			
	R. euleeides	1	20			
nad4	*D. ambulator*	1	2	1 (15)	0	15
	D. papillatum	1	2	1 (29)	7	22
	D. sp. 2	1	2	1 (14)	0	14
	R. euleeides	1	2	1 (14)	1	13
nad5	*D. ambulator*	2	7 + 25			
	D. papillatum	0	0			
	D. sp. 2	2	7 + 1			
	R. euleeides	2	6 + 26			
nad7	*D. ambulator*			1 (4)	2	2
	D. papillatum			1 (1)	1	0
	D. sp. 2			2 (4)	1 + 2	1 + 0
	R. euleeides			2 (5)	2 + 0	2 + 1
nad8	*D. ambulator*					
	D. papillatum					
	D. sp. 2					
	R. euleeides					
rnl	*D. ambulator*	1	26			
	D. papillatum	1	26			
	D. sp. 2	1	25			
	R. euleeides	1	30			
rns	*D. ambulator*	1	9	1 (46)	22	24
	D. papillatum	1	8	1 (45)	15	30
	D. sp. 2	1	5	1 (24)	11	13
	R. euleeides	1	9	1 (47)	33	14
y1	*D. ambulator*	1	8	0	0	0
	D. papillatum	1	4	1 (11)	4	7
	D. sp. 2	1	2	1 (13)	5	8
	R. euleeides	0	0	0	0	0
y2	*D. ambulator*	1	18	1 (5)	4	1

Gene	Species	Nr. of U-appendage sites [a]	U-tract length [nt]	Nr. of substitution clusters (Total edits)	Nr. of A-to-I sites	Nr. of C-to-U sites
	D. papillatum	2	18 + 11	1 (3)	1	2
	D. sp. 2	2	19 + 5	1 (9)	8	1
	R. euleeides	2	18 + 6	1 (3)	2	1
y3	*D. ambulator*	3	27 + 15 + 3	0	0	0
	D. papillatum	3	27 + 16 + 1	1 (7)	1	6
	D. sp. 2	3	28 + 16 + 7	0	0	0
	R. euleeides	3	27 + 14 + 2	1 (7)	1	6
y5	*D. ambulator*	1	50	1 (14)	0	14
	D. papillatum	1	50	1 (18)	0	18
	D. sp. 2	1	50	1 (14)	4	10
	R. euleeides	2	48 + 2	1 (22)	0	22
y6	*D. ambulator*	n.d.	n.d.			
	D. papillatum	1	6			
	D. sp. 2	n.d.	n.d.			
	R. euleeides	n.d.	n.d.			
**Total**	*D. ambulator*	15	216	5 (84)	28	56
	D. papillatum	16*	198*	7 (114)	29	85
	D. sp. 2	16	196	7 (78)	31	47
	R. euleeides	17	225	7 (98)	39	59

[a] Asterisks indicate that U-appendage sites in the *y4* gene of *D. papillatum* (two sites; 28 nt and 12 nt long) were not taken into account, because the gene was not found in the other three species. n.d., not determined

Supplementary Table S4. Oligonucleotides used in this study.

Oligonucleotide	Sequence (5′ → 3′)	Target	Application
CDS-III	ATTCTAGAGGCCGAGGCGGCCGACATG(T)$_{30}$VN	poly-(A) RNAs	RT
CDS-III-ter	ATTCTAGAGGCCGAGGCG	poly-(A) RNAs	PCR
dp123	GGCTTATCGGCGAAATTCACTTTCTG	dp124 (RNA)	5′-RACE
dp124 (RNA)	rGrGrCrUrUrArUrCrGrGrCrGrArArArUrU rCrArCrUrUrUrCrUrG	5′ phosphorylated RNAs	5′-RACE
dp301	TGTGGTGTACTGCTGGTATCAG	*Dp_y5-m1*	PCR
dp302	CATAGCGTAGGTGCTCCGTAG	*Dp_y5-m3*	PCR
dp303	GTTGTGGGTGCGTTAGTAGCA	*Dp_y5-m1*	PCR
dp304	CATGGGATGTATGTACCGGG	*Dp_y5-m2*	PCR
da25	GGCATAGAACTGCAGCTACCTAT	*Da*_C05 chromosome	PCR
da26	ATACATGGTAGCACACATGC	*Da*_C05 chromosome	PCR
da39	GTGTAGAGGATAGCCATACATGCATT	*Da_rns-m1*	primer extension
da42	TCGTAGCGATACATGCTCCTGT	*Da_y5-m2*	PCR
da43	CTACTGCTAGTACGCTGTCTGCTA	*Da_nad5-m9*	PCR
da44	GTAGTAGTAGAGGACAGCATAGCG	*Da_y5-m3*	PCR
da45	ACTACAGCTACTGCCATGTGTG	*Da_y5-m1*	PCR
da46	CCACAGCGTATATGCACTGCT	*Da*_A02 chromosome	PCR
da47	TGGTAGGTGATGCCTGTGCT	*Da*_A02 chromosome	PCR
da48	CCAACAGAGGCTAGACGTGTG	*Da_nad7-m5*	PCR
da49	TCTAGTGCTAGGCATACAGGGAC	*Da_nad7-m5*	PCR
da50	GTAGCACAGACAGCAGGGCT	*Da_y3-m4*	PCR
da51	CATTGCTCTCCCGACATCCA	*Da_cox1-m5*	PCR
da52	GTGAGCCGAATCTTTGGAGG	*Da*_U04 chromosome	PCR
da53	TAAGGGTTGGTGTATGCCTAGTG	*Da_y3-m3*	PCR
da54	TGCTTCTGTGAGTCATGCCTGT	*Da_nad4-m3*	PCR
da55	GCGTTTATGCAGCAGTAG	*Da_y3-m5*	PCR
da56	ACGCAGCACACGCTCTCC	*Da_nad5-m10*	PCR
da59	TGGAGCCATCATGTGTGCC	*Da*_c2-cassette downstream	PCR
da60	GGAGAGCGTGTGCTGCGT	*Da_nad5-m10*	PCR
da61	GTGTCCGTCTGCTGATGGAAT	*Da*_c2-cassette upstream	PCR
ds52	GAAGCATGTCGGACAGTAAGGA	*Ds_y3-m2*	RT-PCR
ds53	TGCGGACTGTGCTATGATAGTGT	*Ds_y5-m1*	PCR
ds54	TGGACAGGAGCAAGACATCAGT	*Ds_y5-m2*	PCR
ds55	GACTTAGGCAGCGTTGTCTTC	*Ds_nad1-m4*	PCR
re51	GGTATCCGCACTAGCACCATCA	*Re_y3-m4*	PCR
re52	GCCAATGATGCAACGCACACTA	*Re_y3-m2*	RT-PCR
re54	CCACTGGGGATACACATACCATC	*Re_y2-m3*	PCR
re55	GCACTCCTGATATGCTAACAGCTATG	*Re_y5-m1*	PCR
re56	TGCTATGCAGGGTGATGATGA	*Re_nad5-m9*	PCR
re57	TTTTTTTTTTTTAGTACTAGCAG	*Re_y2-m4*	sequencing
re58	CCTGCTGATGTGTGTATCTCTGTG	*Re_y5-m3*	PCR
re59	ATCATAGTATCTGGTGGCGGA	*Re_y5-m2*	PCR
re60	CTCTGATGGTATCACCTGTTGAC	*Re_nad1-m4*	PCR
re61	AAGCAATGCCAGCTATCCGT	*Re_nad4-m6/m7*	PCR
re63	CATGCTGTAGAGGCACCAAAGG	*Re_nad7-m8*	PCR

Supplementary Table S5. Sequencing libraries used in this study.

Library name	Material	Library prep kit (approx. insert size)	Sequencing technology [a]	Nr. of raw read pairs	Read length [nt]	SRA ID
Da	*D. ambulator* total DNA	TruSeq DNA (0.5 kb)	MiSeq PE	4,712,258	300	SRR5998381
Ds	*D.* sp. 2 total DNA	TruSeq DNA (0.5 kb)	MiSeq PE	4,207,560	300	SRR5998377
Re	*R. euleeides* total DNA	TruSeq DNA (0.5 kb)	MiSeq PE	4,990,130	300	SRR5998374
DaTs	*D. ambulator* total RNA	TruSeq small RNA (0.02-0.5 kb)	HiSeq PE	24,051,949	150	SRR5998380
DaT	*D. ambulator* total RNA	TruSeq RNA (0.2 kb)	MiSeq PE SS	3,038,621	250	SRR5998379
DaT-Hi	*D. ambulator* total RNA	TruSeq RNA (0.2 kb)	HiSeq PE SS	15,789,289	150	SRR5998378
DsT	*D.* sp. 2 total RNA	TruSeq RNA (0.2 kb)	MiSeq PE SS	3,150,711	250	SRR5998376
DsT-Hi	*D.* sp. 2 total RNA	TruSeq RNA (0.2 kb)	HiSeq PE SS	17,017,840	150	SRR5998375
ReT	*R. euleeides* total RNA	TruSeq RNA (0.2 kb)	MiSeq PE SS	3,385,789	250	SRR5998383
ReT-Hi	*R. euleeides* total RNA	TruSeq RNA (0.2 kb)	HiSeq PE SS	15,613,531	150	SRR5998382

[a] PE, paired-end; SS, strand-specific.

Supplementary Table S6. Ambiguity of junction positions in *D/R* diplonemids due to the presence of identical nucleotides at the 3′ end of the upstream module and the 5′ end of the downstream module.

Gene	Junction		Ambiguous in [nr. of species]	Length of identical sequence [a]			
	Upstream module	Downstream module		*Da*	*Dp*	*Ds*	*Re*
atp6	m1	m2	2	0 nt	0 nt	1 nt	2 nt
atp6	m2	m3	1	0 nt	1 nt	0 nt	0 nt
cob	m1	m2	2	1 nt	0 nt	2 nt	0 nt
cob	m2	m3	2	1 nt	1 nt	0 nt	0 nt
cob	m3	m4	3	2 nt	0 nt	1 nt	2 nt
cob	m4	m5	2	1 nt	0 nt	1 nt	0 nt
cob	m5	m6	1	0 nt	0 nt	1 nt	0 nt
cox1	m1	m2	3	0 nt	1 nt	2 nt	1 nt
cox1	m2	m3	3	2 nt	1 nt	0 nt	1 nt
cox1	m3	m4	2	2 nt	0 nt	0 nt	1 nt
cox1	m4	m5	1	0 nt	0 nt	3 nt	0 nt
cox1	m5	m6	3	3 nt	2 nt	0 nt	2 nt
cox1	m6	m7	0	0 nt	0 nt	0 nt	0 nt
cox1	m7	m8	2	0 nt	1 nt	1 nt	0 nt
cox1	m8	m9	3	2 nt	2 nt	0 nt	3 nt
cox2	m1	m2	1	0 nt	4 nt	0 nt	0 nt
cox2	m2	m3	2	1 nt	1 nt	0 nt	0 nt
cox2	m3	m4	1	0 nt	0 nt	0 nt	1 nt
cox3	m1	m2	2	2 nt	0 nt	0 nt	3 nt
cox3	m2	m3	2	0 nt	3 nt	0 nt	4 nt
nad1	m1	m2	3	0 nt	2 nt	3 nt	3 nt
nad1	m2	m3	1	2 nt	0 nt	0 nt	0 nt
nad1	m3	m4	3	1 nt	5 nt	0 nt	1 nt
nad1	m4	m5	1	0 nt	1 nt	0 nt	0 nt
nad4	m1	m2	2	0 nt	1 nt	0 nt	6 nt
nad4	m2	m3	2	1 nt	1 nt	0 nt	0 nt
nad4	m3	m4	1	2 nt	0 nt	0 nt	0 nt
nad4	m4	m5	1	0 nt	0 nt	0 nt	2 nt
nad4	m5	m6	1	0 nt	0 nt	0 nt	1 nt
nad4	m6	m7	0	0 nt	0 nt	0 nt	0 nt
nad4	m7	m8	2	1 nt	0 nt	1 nt	0 nt
nad5	m1	m2	3	0 nt	1 nt	2 nt	1 nt
nad5	m2	m3	2	1 nt	0 nt	4 nt	0 nt
nad5	m3	m4	2	0 nt	0 nt	1 nt	1 nt
nad5	m4	m5	1	4 nt	0 nt	0 nt	0 nt
nad5	m5	m6	0	0 nt	0 nt	0 nt	0 nt
nad5	m6	m7	2	0 nt	1 nt	1 nt	0 nt
nad5	m7	m8	2	0 nt	0 nt	2 nt	1 nt
nad5	m8	m9	2	3 nt	0 nt	0 nt	3 nt
nad5	m9	m10	3	1 nt	3 nt	0 nt	2 nt
nad5	m10	m11	1	0 nt	0 nt	0 nt	2 nt
nad7	m1	m2	3	0 nt	1 nt	1 nt	1 nt
nad7	m2	m3	1	0 nt	0 nt	0 nt	3 nt
nad7	m3	m4	1	0 nt	0 nt	0 nt	1 nt
nad7	m4	m5	2	0 nt	0 nt	2 nt	1 nt
nad7	m5	m6	1	0 nt	1 nt	0 nt	0 nt
nad7	m6	m7	2	1 nt	0 nt	0 nt	1 nt
nad7	m7	m8	1	1 nt	0 nt	0 nt	0 nt
nad7	m8	m9	3	2 nt	3 nt	0 nt	1 nt
nad8	m1	m2	0	0 nt	0 nt	0 nt	0 nt
nad8	m2	m3	2	2 nt	1 nt	0 nt	0 nt
rnl	m1	m2	4	1 nt	2 nt	2 nt *	1 nt *

Gene	Junction		Ambiguous in [nr. of species]	Length of identical sequence [a]			
	Upstream module	Downstream module		*Da*	*Dp*	*Ds*	*Re*
y1	m1	m2	0	0 nt	0 nt	0 nt	0 nt
y2	m1	m2	3	1 nt	0 nt	2 nt	3 nt
y2	m2	m3	2	1 nt	1 nt	0 nt	0 nt
y2	m3	m4	4	1 nt	1 nt *	1 nt	1 nt *
y3	m1	m2	1	0 nt	0 nt	n.a.	2 nt
y3	m2	m3	1	0 nt	1 nt	0 nt	0 nt
y3	m3	m4	2	1 nt	0 nt	0 nt	1 nt *
y3	m4	m5	4	3 nt *	1 nt *	1 nt	1 nt *
y5	m1	m2	3	1 nt *	0 nt	2 nt *	1 nt *
y5	m2	m3	1	1 nt	0 nt	0 nt	0 nt
y6	m1	m2	1	0 nt	0 nt	2 nt	0 nt

**Summary — Number of junctions**	**All four species**	***Da***	***Dp***	***Ds***	***Re***
Unambiguous	136	32	36	39	29
Initially ambiguous due to overlaps	115	31	27	23	34
Remain ambiguous	11	2	2	2	5
All	251	63	63	62	63

[a] Asterisks indicate junctions that remained unresolved after RNA-Seq-based curation. n.a., not applicable (junction absent)

Supplementary Table S7. GenBank accession numbers of edited mitochondrial RNAs of *D/R*-clade diplonemids.

Organism	RNA	GenBank Acc. Nr.	Reference
D. ambulator	*atp6*	MF436931	This report
	cob	MF436932	This report
	cox1	MF436933	This report
	cox2	MF436934	This report
	cox3	MF436935	This report
	nad1	MF436936	This report
	nad4	MF436937, KU341385	This report, Moreira *et al*., 2016
	nad5	MF436938	This report
	nad7	MF436939	This report
	nad8	MF436940	This report
	rnl	MF436941	This report
	rns	MF436942	This report
	y1	MF436943	This report
	y2	MF436944	This report
	y3	MF436945	This report
	y5	MF436946	This report
	y6	MF436947	This report
D. sp. 2	*atp6*	MF436948	This report
	cob	MF436949	This report
	cox1	MF436950	This report
	cox2	MF436951	This report
	cox3	MF436952	This report
	nad1	MF436953	This report
	nad4	MF436954, KU341387	This report, Moreira *et al*., 2016
	nad5	MF436955	This report
	nad7	MF436956	This report
	nad8	MF436957	This report
	rnl	MF436964	This report
	rns	MF436963	This report
	y1	MF436958	This report
	y2	MF436959	This report
	y3	MF436960	This report
	y5	MF436961	This report
	y6	MF436962	This report
R. euleeides	*atp6*	MF436965	This report
	cob	MF436978	This report
	cox1	MF436979	This report
	cox2	MF436966	This report
	cox3	MF436967	This report
	nad1	MF436968	This report
	nad4	MF436969, KU341389	This report, Moreira *et al*., 2016
	nad5	MF436970	This report
	nad7	MF436971	This report
	nad8	MF436972	This report
	rnl	MF436981	This report
	rns	MF436980	This report
	y1	MF436973	This report
	y2	MF436974	This report
	y3	MF436975	This report
	y5	MF436976	This report
	y6	MF436977	This report
D. papillatum	*atp6*	KU341361	Moreira *et al*., 2016
	cob	KU341362	Moreira *et al*., 2016

**Organism**	**RNA**	**GenBank Acc. Nr.**	**Reference**
	cox1	KU341363	Moreira *et al*., 2016
	cox2	KU341364	Moreira *et al*., 2016
	cox3	KU341365	Moreira *et al*., 2016
	nad1	KU341366	Moreira *et al*., 2016
	nad4	KU341367	Moreira *et al*., 2016
	nad5	KU341368	Moreira *et al*., 2016
	nad7	KU341369	Moreira *et al*., 2016
	nad8	KU341370	Moreira *et al*., 2016
	rnl	KU341371	Moreira *et al*., 2016
	rns	KU341372	Moreira *et al*., 2016
	y1	KU341373	Moreira *et al*., 2016
	y2	KU341374	Moreira *et al*., 2016
	y3	KU341375	Moreira *et al*., 2016
	y4	KU341376	Moreira *et al*., 2016
	y5	KU341377	Moreira *et al*., 2016
	y6	KU341378	Moreira *et al*., 2016

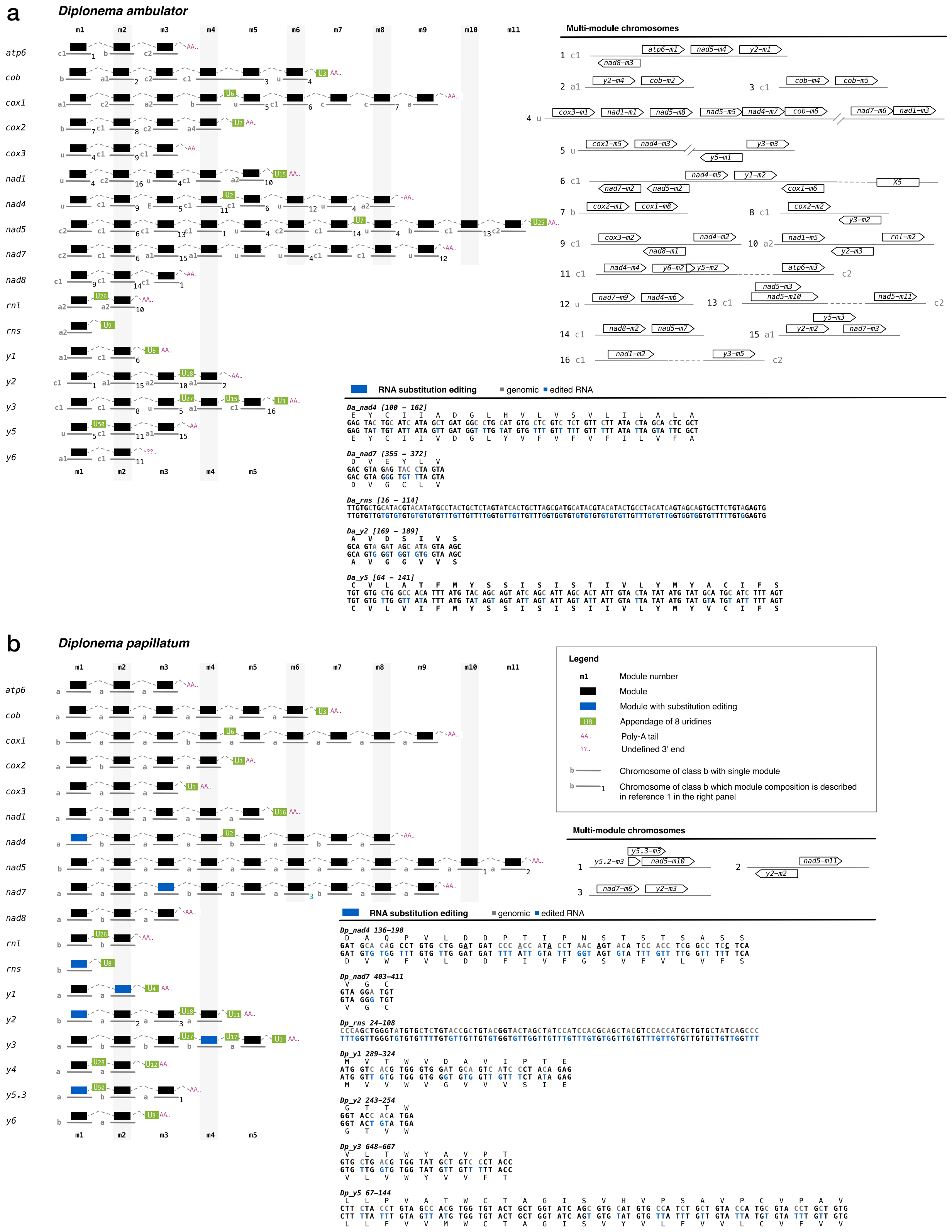

Supplementary Fig. S1. Schematic representation of the coding content of diplonemid mtDNAs. Transcripts and their module composition are listed in the left panel. Module transcripts edited by substitution are coloured in blue, and appendage of uridines is represented by a green box. Multi-module

chromosomes are listed in the top-right panel. The bottom-right panel shows the genomic and the corresponding fully edited cDNA of module regions that undergo substitution editing, together with codon assignments. Editing sites are highlighted in grey in the genomic, and in blue in the cDNA sequence. (**a**) *Diplonema ambulator*, (**b**) *Diplonema papillatum*, (**c**) *Diplonema* sp. 2, (**d**) *Rhynchopus euleeides*.

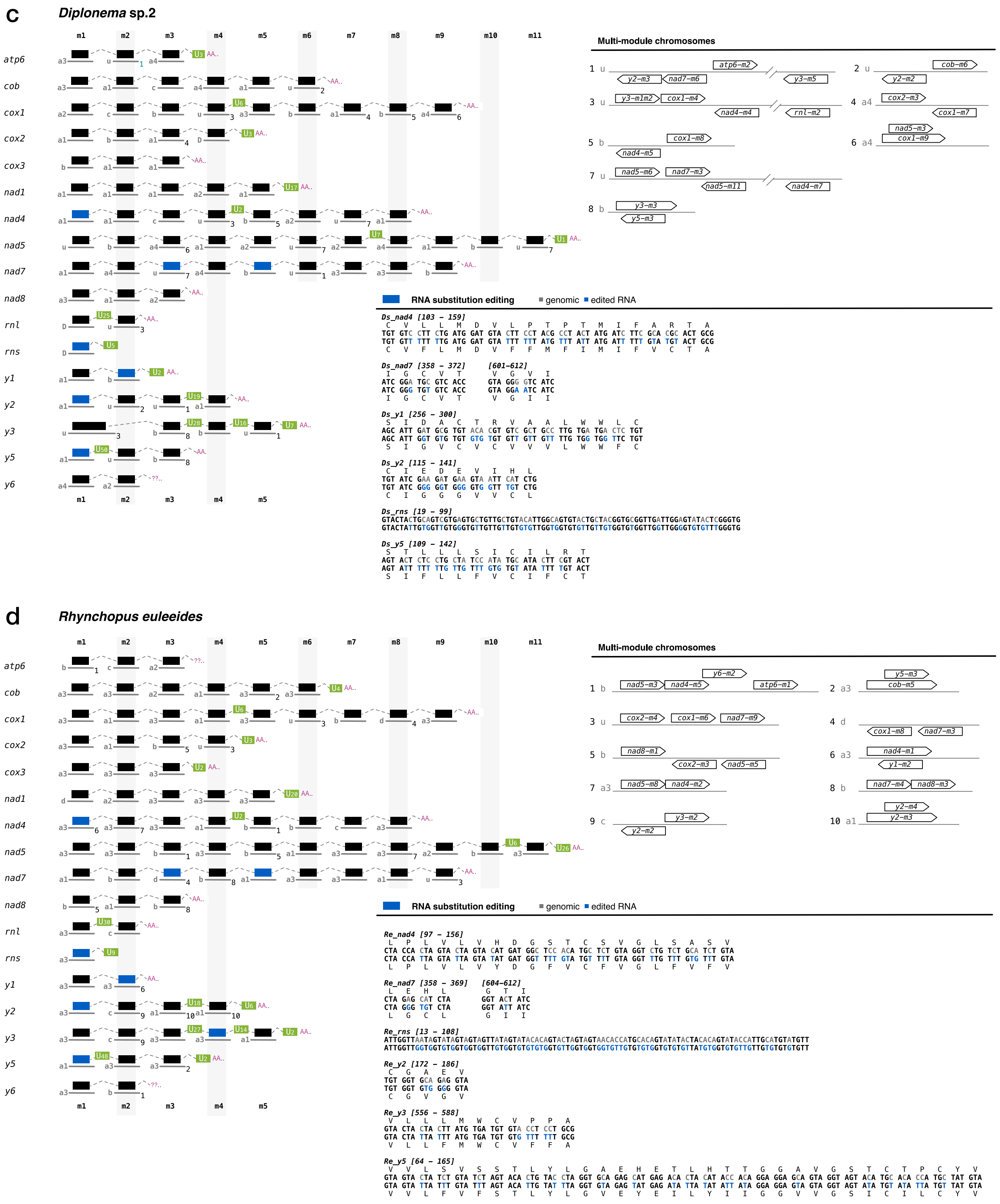

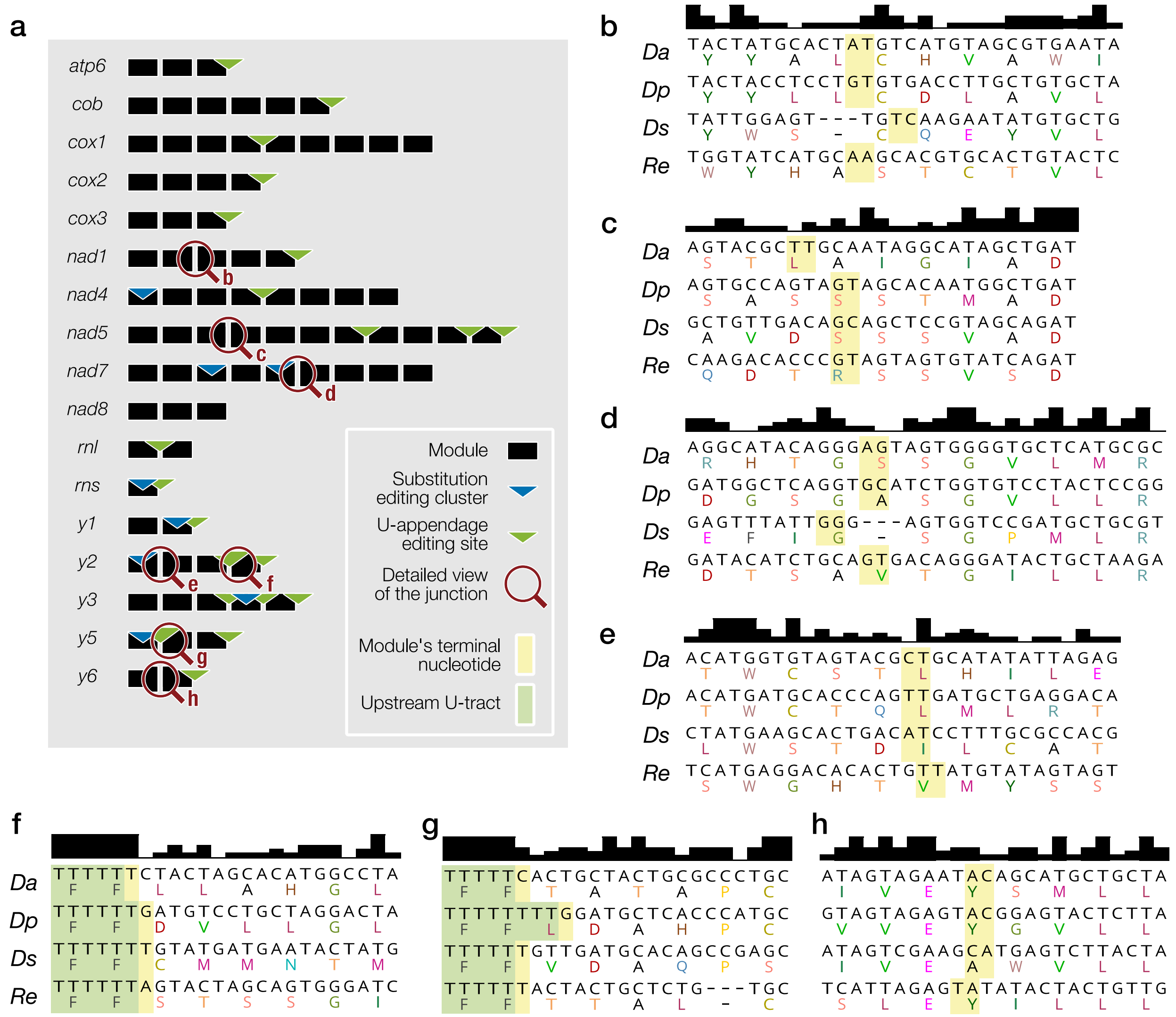

Supplementary Fig. S2. Variations in mitochondrial gene breakpoints across *D/R*-clade diplonemids. (**a**) Overview of gene fragmentation and positions of RNA editing sites. Inset, the colour-shading key and the feature key: black rectangles, modules; green triangles, U-appendage RNA editing sites at 3′ ends of modules; blue triangles, substitution clusters; the magnification glass symbol and letter refer to the sequence-level close-up in **b-h**. (**b-h**) Sections of multiple alignments of cDNA sequences with focus on a particular junction. The upper track indicates by shading and bar height the extent of DNA sequence similarity (see Methods for details). The inferred protein sequence is shown below in the one-letter code. *Da*, *D. ambulator*; *Dp*, *D. papillatum*; *Ds*, *D.* sp. 2; *Re*, *R. euleeides*. (**b**) Detail of *nad1*-m2 and -m3. (**c**) *nad5*-m3 and -m4. (**d**) *nad7*-m5 and -m6. (**e**) *y2*-m1 and -m2. (**f**) U-tract appended to *y2*-m3 followed by -m4. (**g**) U-tract appended to *y5*-m1 followed by -m2. (**h**) *y6*-m1 and -m2. Note that the module junctions *Re_y2-m1/m2* (**e**), *Da_y2-m3/m4* (**f**), and *Re_y6-m1/m2* (**h**) are shifted by 1 nt. These are the only instances, where the size differences between homologous modules are not a multiple of three.

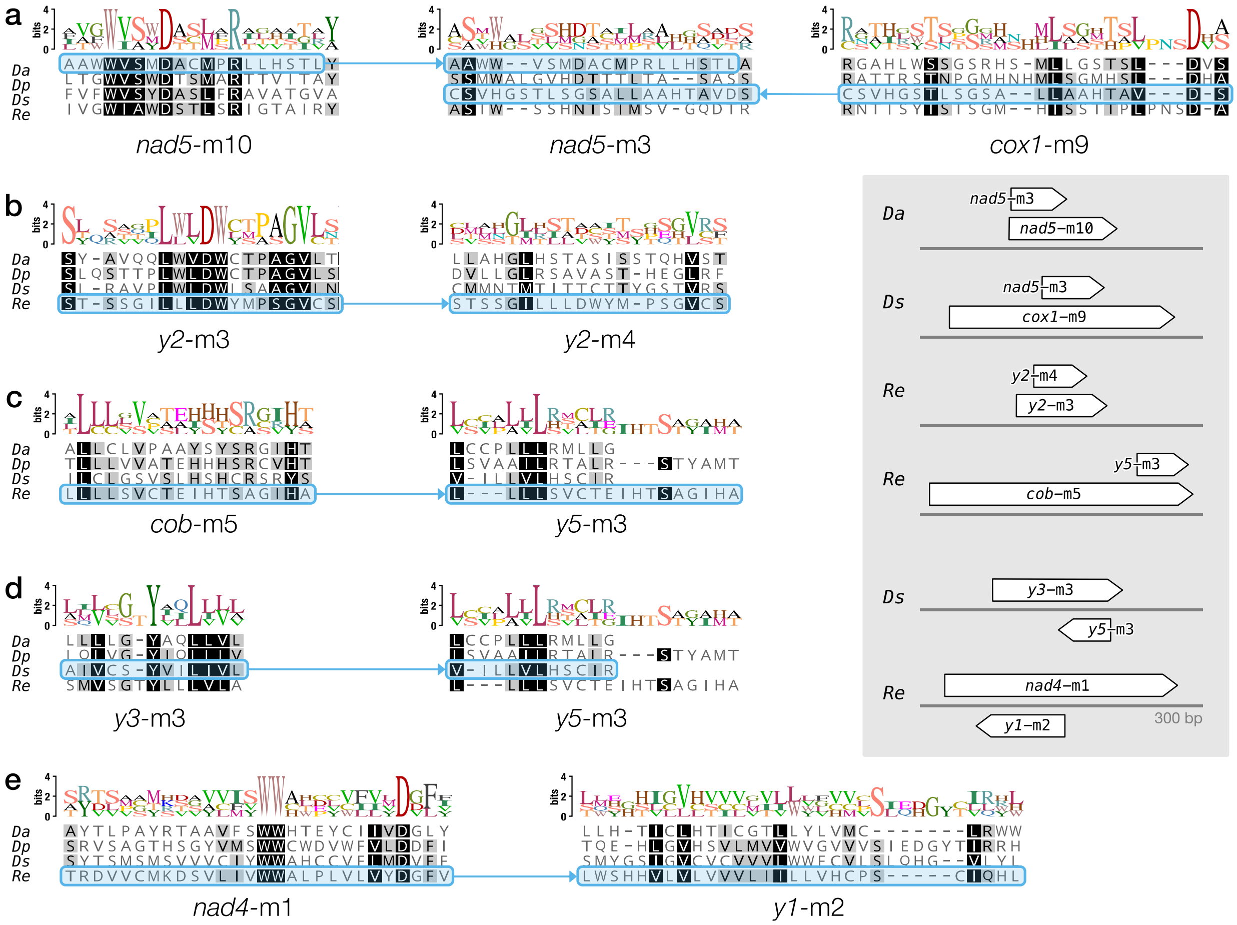

Supplementary Fig. S3. Multiple protein-sequence alignments of nested modules. The embedded modules code for more divergent protein regions than the enclosing modules. Blue-boxed sequences derive from the same nucleotide sequence. The arrow points towards the embedded module. Note that for the enclosing module, only the region that overlaps the embedded module is shown. (**a**) Part of *nad5*-m10 and *cox1*-m9 is reused as *nad5*-m3 in *D. ambulator* and *D.* sp. 2, respectively. (**b, c**) In *R. euleeides*, part of *y2*-m3 is reused as *y2*-m4 and part of *cob*-m5 as *y3*-m5. (**d**) In *D.* sp. 2, *y5*-m3 is encoded by the reverse complementary strand inside *y3*-m3. (**e**) In *R. euleeides*, *y1*-m2 is located on the reverse complementary strand inside *nad4*-m1. Inset, genomic arrangement of the nested modules (to scale with the grey bar representing 300 bp). *Da*, *Diplonema ambulator*; *Dp*, *Diplonema papillatum*; *Ds*, *Diplonema* sp. 2; *Re*, *Rhynchopus euleeides*.

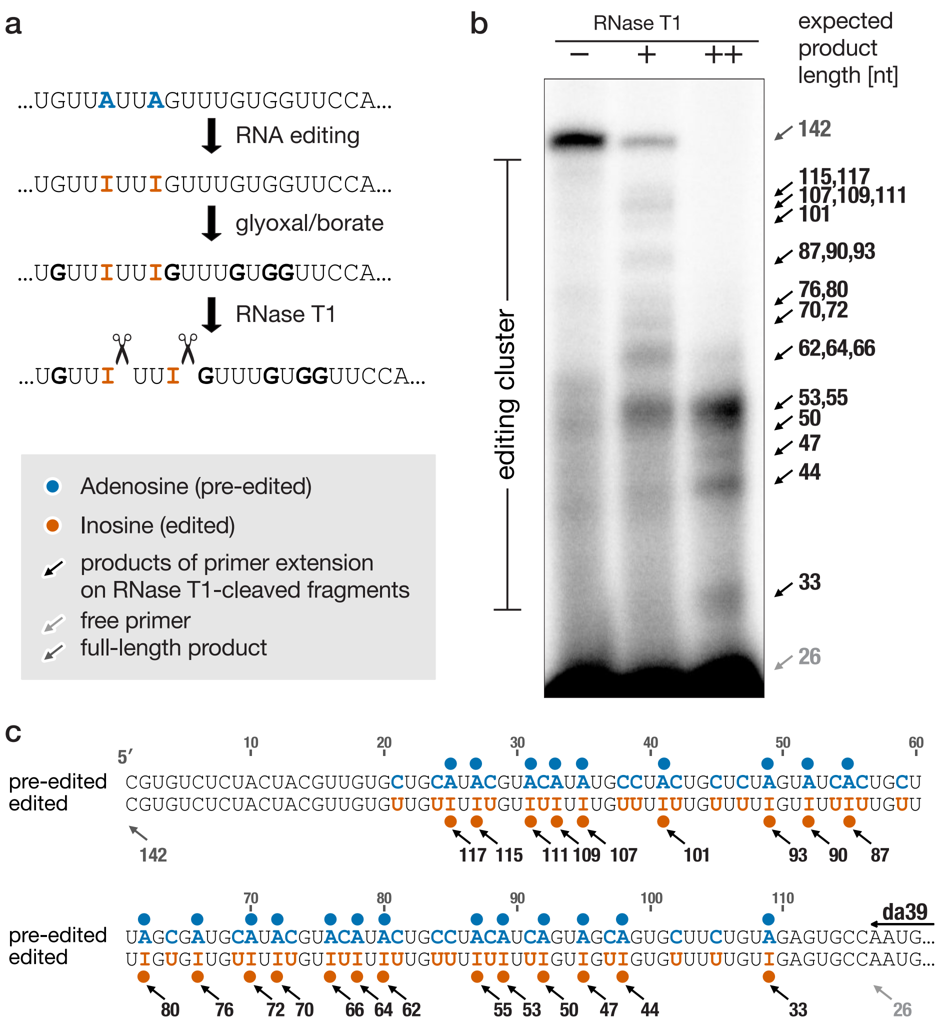

Supplementary Fig. S4. Experimental confirmation of deamination RNA editing of mt-SSU rRNA from *D. ambulator*. (**a**) The experimental approach involving glyoxal/borate- and RNase T1-treatment of transcripts. The inset shows the symbols that indicate editing sites and products of reverse transcription (RT). (**b**) Primer extension of mt-SSU rRNA to map RNase T1 cleavage sites at inosines using total RNA as a template. Lanes: −, untreated template; +, ++, digestion with 10 U and 50 U RNase T1, respectively, allowing detection of cleavage intermediates. Bands represent RT-stops one nucleotide prior to an I. The expected product sizes are indicated on the right. (**c**) The sequence schema of pre-edited and edited *Da_rns* illustrates the positions of predicted Is, as well as the expected sizes of RT products.

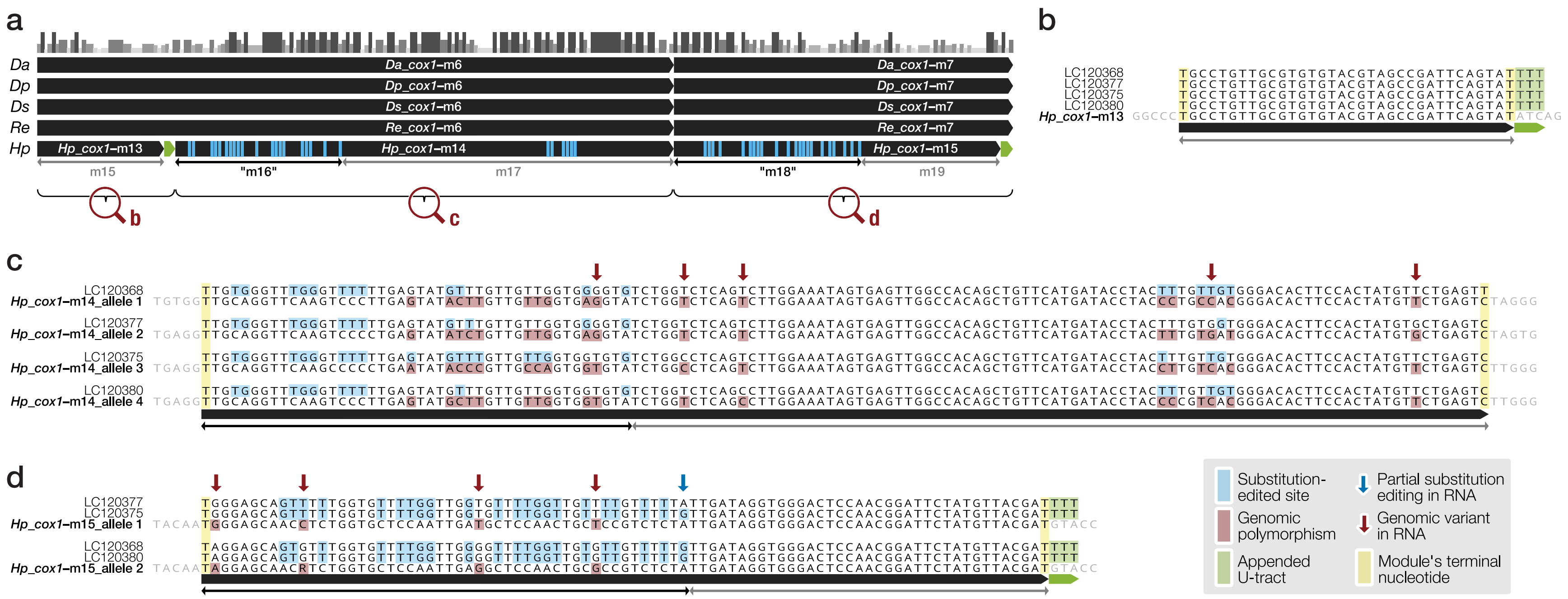

Supplementary Fig. S5. Numerous massive substitution editing clusters in *cox1* of *Hemistasia phaeocysticola* hinder identification of modules in the mtDNA sequence. (**a**) Overview of a segment of the multiple alignment of *cox1* transcripts from *Diplonema ambulator* (*Da*), *D. papillatum* (*Dp*), *D.* sp. 2 (*Ds*), *Rhynchopus euleeides* (*Re*), and *Hemistasia phaeocysticola* (*Hp*). The upper track indicates the similarity among the five sequences by bar shading and height. The region corresponds to modules 6 and 7 in *D/R* diplonemids and to modules 13, 14, and 15 (numbering according to our re-annotation) of *H. phaeocysticola*. In the original report [2], this region was referred to as m15 to m19, with "m16" and "m18" corresponding to modules "missing" in the mtDNA sequence (see the highlighting by black and grey horizontal bars below the alignment). The blue vertical bars indicate the substitution RNA editing sites inferred from the comparison of the genomic and transcript data as detailed below. The magnification glass symbols and letters refer to sequence-level close-ups of mtDNA and cDNA comparisons from *H. phaeocysticola* in **b-d**. (**b**) Detailed view of *Hp_cox1-m13* from mtDNA and four representative cDNAs (GenBank Acc. LC120368, LC120377, LC120375, LC120380). Flanking non-coding sequence is indicated in light grey. (**c**) Detailed view of the four alleles of *Hp_cox1-m14* from mtDNA and four representative cDNAs (same as above), each corresponding to one of the genomic variants. Diagnostic positions are indicated by red arrows above the alignment. Note that substitution RNA editing also makes the various mitochondrial genomic alleles of modules more similar to each other. (**d**) Detailed view of the two alleles of *Hp_cox1-m15* from mtDNA and four representative cDNAs (same as above). Inset, the colour-shading key for feature highlighting.

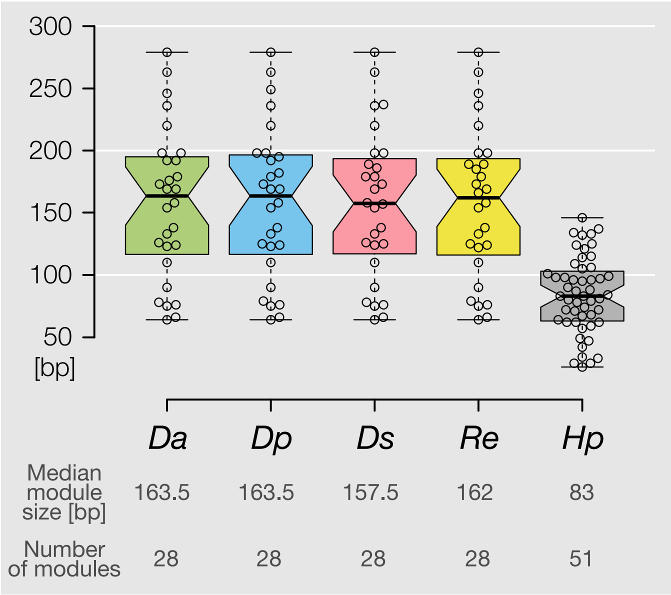

Supplementary Fig. S6. The average module size in *Hemistasia* is half their size of those in *D/R* diplonemids. Size distributions were compared for gene modules of *cob*, *cox1*, *cox2*, and *nad7* from *Diplonema ambulator* (*Da*), *D. papillatum* (*Dp*), *D.* sp. 2 (*Ds*), *Rhynchopus euleeides* (*Re*), and *Hemistasia phaeocysticola* (*Hp*). In the latter species, values correspond to our data re-analysis (see main text and **Supplementary Fig. S5, S7**).

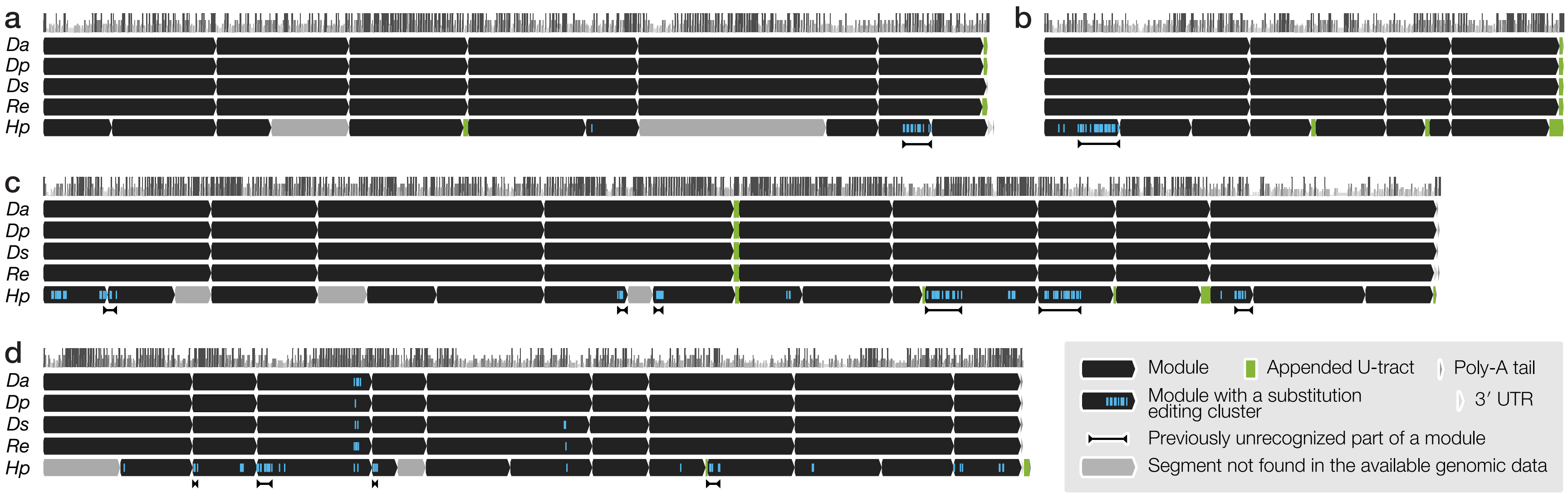

Supplementary Fig. S7. Additional substitution RNA editing clusters and U-appendage sites occur near additional modules junctions in *Hemistasia phaeocysticola*. Comparison of gene fragmentation and editing sites in the mature transcripts of the genes *cob* (**a**), *cox2* (**b**), *cox1* (**c**), and *nad7* (**d**) from *Diplonema ambulator* (*Da*), *D. papillatum* (*Dp*), *D.* sp. 2 (*Ds*), *Rhynchopus euleeides* (*Re*), and *H. phaeocysticola* (*Hp*). The track above the multiple sequence alignment indicates the similarity among the five sequences by bar shading and height. Inset, the feature key.

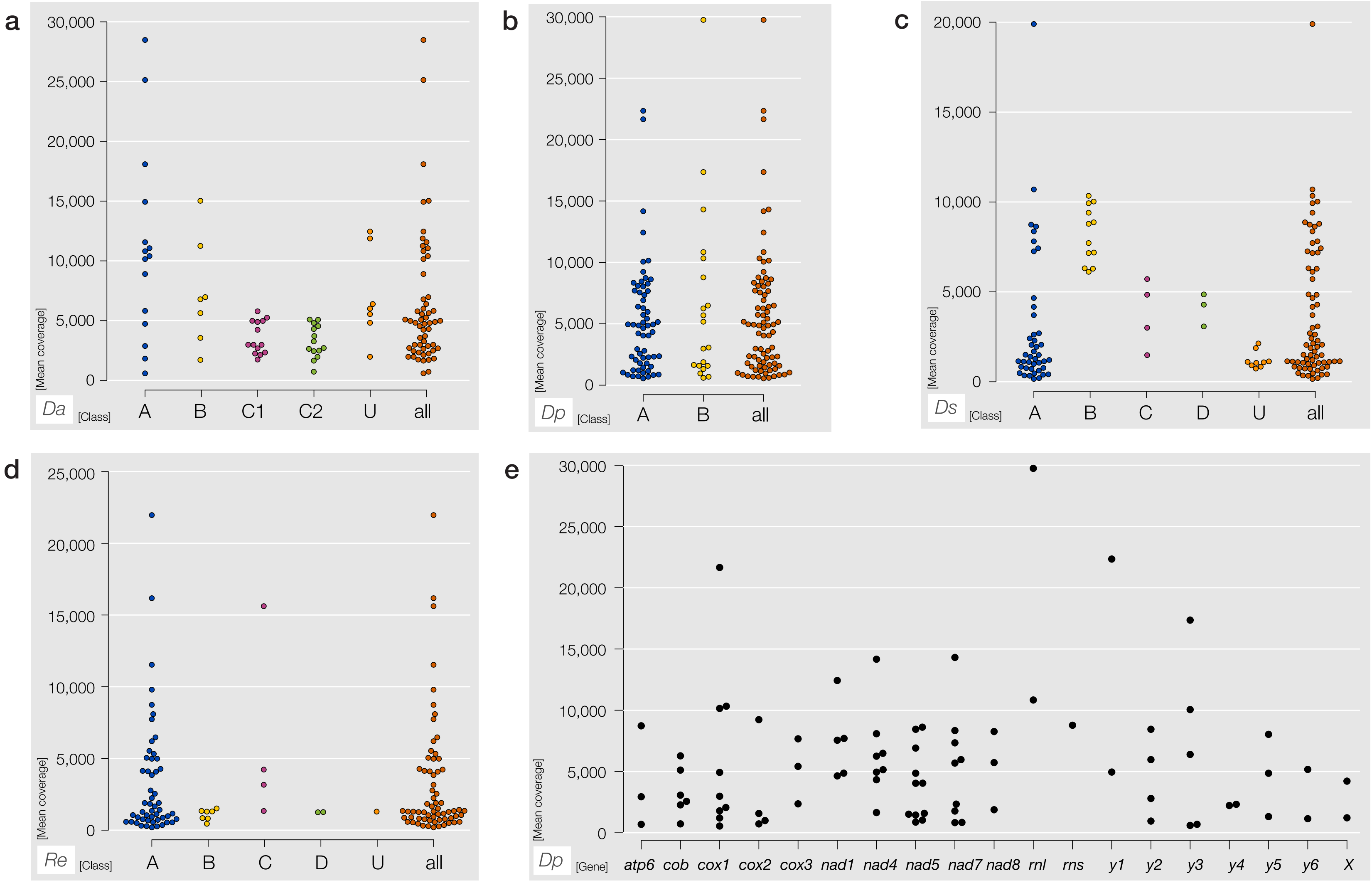

Supplementary Fig. S8. Fluctuation of mitochondrial chromosome abundance in *D/R*-group diplonemids. (**a-d**) Distribution of read mean coverage of individual cassettes or module arrays binned by class (A-D, U, and all combined). (**a**) Distribution for *D. ambulator* (*Da*). Note that C1 and C2 classes, which constitute bi-cassette chromosomes, are plotted separately to show similar coverage, further indicating their association. (**b**) Distribution for *D. papillatum* (*Dp*); (**c**) for *D.* sp. 2 (*Ds*); and (**d**) for *R. euleeides* (*Re*). (**e**) Distribution of read mean coverage of cassettes depending on the gene, whose modules they code for in *D. papillatum* (*Dp*). (For details, see also **Supplementary Table S2** and Methods; *D. papillatum* data are from [1].)

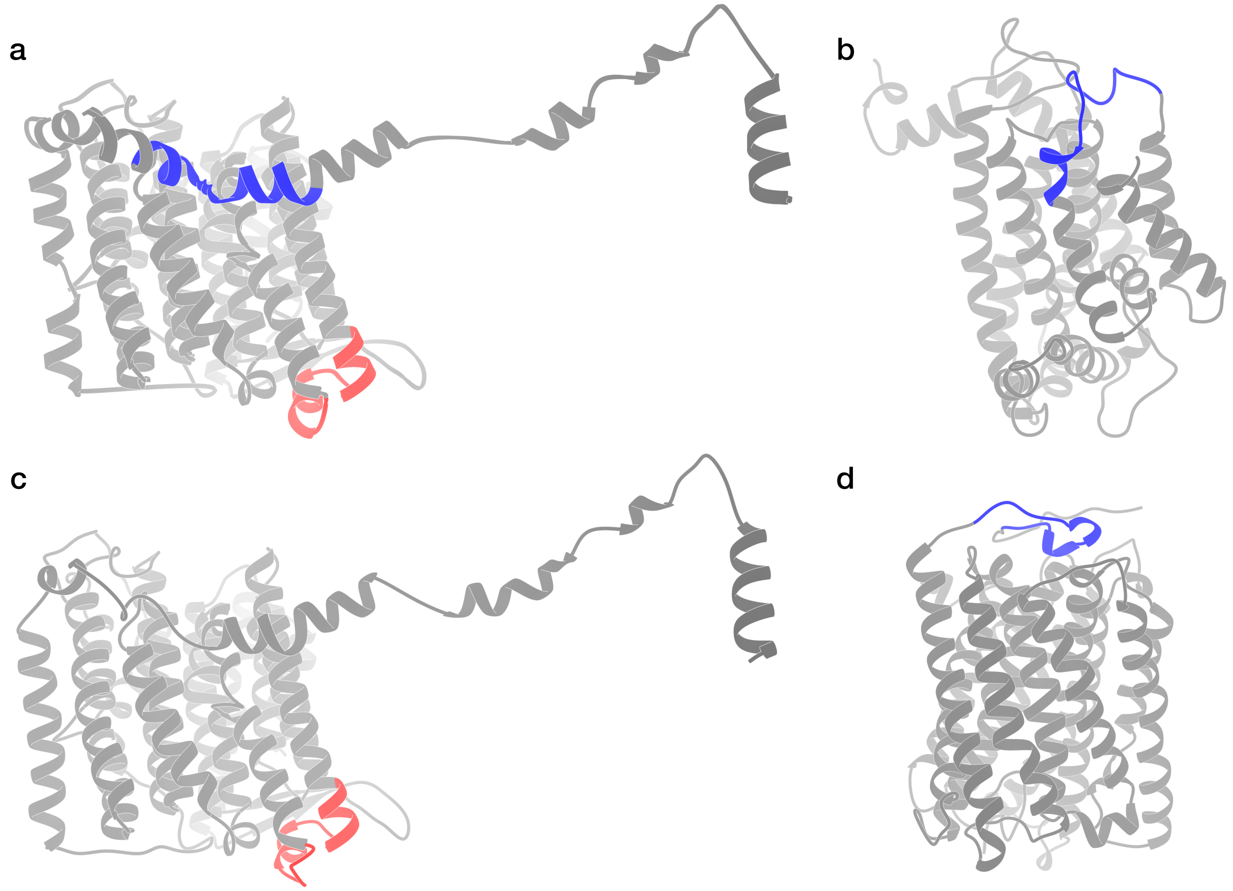

Supplementary Fig. S9. Dual-use segments of diplonemid mitochondrial proteins are predicted to specify peripheral loops and short α-helices that connect trans-membrane helices. Segments originating from the enclosing and embedded module are shown in blue and orange, respectively. The C-terminus of the three-dimensional protein models is on the right side. (**a**) Nad5 protein from *D. ambulator* (*nad5*-m3 is embedded in *nad5*-m10). (**b**) Cob protein from *R. euleeides* (*y5*-m3 is embedded in *cob*-m5). Note that only the segment of the enclosing module is highlighted, since the *y5* gene is unassigned. (**c**, **d**) Nad5 and Cox1 proteins from *D.* sp. 2, respectively (*nad5*-m3 is embedded in *cox1*-m9). (For details, see also **Supplementary Fig. S3** and Methods.)

SUPPLEMENTARY REFERENCES

1. Moreira, S., Valach, M., Aoulad-Aissa, M., Otto, C. & Burger, G. Novel modes of RNA editing in mitochondria. *Nucleic Acids Res.* **44,** 4907–4919 (2016).
2. Yabuki, A., Tanifuji, G., Kusaka, C., Takishita, K. & Fujikura, K. Hyper-eccentric structural genes in the mitochondrial genome of the algal parasite *Hemistasia phaeocysticola*. *Genome Biol. Evol.* **8,** 2870–2878 (2016).
